# Energy-efficient wireless sensor networks using unequal clustering and cluster head rotation optimization

**DOI:** 10.1038/s41598-026-56877-9

**Published:** 2026-06-10

**Authors:** Bhaskar Prince, Prabhat Kumar, Sunil Kumar Singh

**Affiliations:** 1 Department of Computer Science and Engineering, National Institute of Technology Patna, Bihar, India; 2 Manipal Institute of Technology Bengaluru, Manipal Academy of Higher Education, Manipal, India

**Keywords:** Wireless sensor network (WSN), Mobile data collector (MDC), Anchor point (AP), Cluster head (CH), Unequal cluster, Computer science, Information technology

## Abstract

The energy hole problem is a significant challenge in wireless sensor networks (WSN) that use multi-hop routing protocols. Nodes near the base station (BS) typically experience higher energy consumption due to higher data traffic, resulting in faster network energy depletion and creating an energy hole near the BS. To address this issue, the paper proposes a solution involving a mobile data collector (MDC) in an unequal grid cluster. The number and size of the clusters are determined based on the radio energy model’s threshold transmission value, which provides balanced data traffic distribution in the network. The cluster head (CH) is elected based on the node’s distance from the cluster centroid and its residual energy. Additionally, the frequency of CH rotation is optimized through an energy-efficient CH change mechanism. The inclusion of an MDC enables data collection from the CHs along the vertical boundaries, effectively reducing the occurrence of energy holes and extending the network’s overall lifespan. Simulation results demonstrate the superior performance of our protocol compared to similar existing schemes. Our proposed work was simulated using OMNeT++, and the results indicate that it achieves approximately 21% less energy consumption than similar existing works.

## Introduction

A Wireless Sensor Network (WSN) comprises small, low-power sensor nodes deployed strategically to detect and measure environmental parameters such as temperature, humidity, and pressure^1,2^. Typically, these networks include a static base station (BS) located either within or outside the deployment area, which serves as the primary data sink for all sensor nodes. In wide-area WSN deployments, nodes usually communicate with the BS using multi-hop transmissions because direct communication over long distances consumes significantly more energy. Multi-hop routing helps conserve energy and enhances the overall efficiency of the protocol. However, it also introduces a significant challenge known as the energy hole problem^3^. The energy hole problem arises when nodes closer to the sink or base station exhaust their energy faster than peripheral nodes due to the disproportionate burden of relaying data. This leads to premature node death in central areas, creating a “hole” that disrupts data transmission paths and significantly reduces overall network performance. Eventually, it disrupts data transmission from other nodes that still have adequate energy. To mitigate this problem, researchers have explored solutions such as (i) unequal clustering, (ii) heterogeneous node deployment, and (iii) the use of mobile elements. This work is the first to combine unequal clustering and Mobile Data Collectors (MDCs) in a unified framework. Among various routing strategies in WSNs, clustering is widely regarded as a highly energy-efficient approach. Nodes are grouped into clusters governed by a cluster head (CH) and selected based on specific criteria^4^. CHs are responsible for gathering data from member nodes, aggregating it, and forwarding it to relay nodes or directly to the BS. The CH role is rotated among member nodes to distribute energy usage evenly. The Low Energy Adaptive Clustering Hierarchy (LEACH) protocol^5^ was the first to propose such a clustering mechanism and demonstrated significant energy savings compared to earlier approaches^6,7^. Nonetheless, LEACH depends on single-hop communication from CHs to the BS, which limits its efficiency in large networks. Later protocols improved this by incorporating multi-hop communication to enhance energy conservation and extend network longevity^8^.

The use of unequal clustering techniques has demonstrated effectiveness in partially addressing the energy hole problem in Wireless Sensor Networks (WSNs)^9,10^. These methods typically recommend creating smaller clusters close to the Base Station (BS) and progressively enlarging the cluster sizes as the distance from the BS increases. This strategy is based on the premise that smaller clusters lead to reduced intra-cluster communication energy losses due to shorter transmission distances and lower data traffic resulting from fewer member nodes. By controlling the maximum cluster size using a distance threshold $D_0$ derived from the first-order radio energy model discussed in Section 3, it becomes feasible to invert this clustering pattern. In expansive network topologies, the frequency at which a cluster member is selected as Cluster Head (CH) becomes a more critical energy factor than the total intra-cluster data traffic. Since CHs typically consume more energy than regular nodes, nodes within smaller clusters are more frequently elected as CHs, leading to faster energy exhaustion. Consequently, forming larger clusters near the BS may prove advantageous in large-scale networks, as it lowers the CH rotation frequency and helps balance energy consumption more effectively. Based on this rationale, we propose employing larger clusters near the BS to reduce the occurrence of energy holes. Although deploying a heterogeneous WSN where nodes differ in energy capacity and capabilities can further alleviate the energy hole problem, such configurations tend to increase the overall cost and operational complexity of the network^11,12^.

In recent advancements, mobile elements have become a vital component in WSNs to enhance data collection efficiency and reduce energy expenditure. Broadly, two primary types of mobile elements are commonly implemented: (i) Mobile Data Collectors (MDCs) and (ii) Mobile Sinks. An MDC operates as a mobile intermediary between sensor nodes and the BS. As it moves along its path, it collects data directly from individual sensor nodes or relay nodes and delivers the aggregated data to the BS upon arrival. In contrast, a mobile sink refers to a BS equipped with mobility. It collects data from the nodes along its trajectory and can communicate directly with external systems when necessary^13,14^. These mobile entities may follow various movement strategies, including random paths, predetermined fixed routes, on-demand movement, and priority-based trajectories. In the present work, we adopt an MDC that traverses a predefined fixed path to collect data specifically from the CHs.

This study proposes an energy-efficient unequal clustering protocol to mitigate the energy hole problem typically observed near the BS. Additionally, the proposed method minimizes the overhead involved in cluster formation, CH selection, and CH role rotation. To ensure balanced load distribution across the network, the protocol employs a grid-based unequal clustering strategy where the maximum size of each cluster is constrained based on a predefined distance threshold. CH selection within each cluster is determined by identifying the node with the shortest transmission distance from the cluster centroid while effectively managing the CH rotation frequency. The protocol also incorporates a dynamic threshold energy level for each cluster, which varies according to the current round number. During the setup phase, a new CH is appointed only if the energy level of the existing CH drops below its designated threshold for that round. This centralized protocol leverages the BS to partition the entire sensing region into hierarchical levels. Each level is then segmented into unequal rectangular sections called grids, which serve as clusters. The selection of the CH is guided by two principal factors: the residual energy of a node and its proximity to the cluster’s centroid. The BS computes the centroid for each cluster and chooses the node closest to it, provided that its residual energy exceeds the average energy of the cluster. Furthermore, integrating an MDC into the protocol enhances energy efficiency by offloading transmission tasks from static nodes. A recent article introduces the Unequal Clustering Energy Hole Avoidance (UCEHA) algorithm to address energy imbalance and hotspot issues in Cognitive Radio Wireless Sensor Networks (CRWSNs)^15^. UCEHA forms unequal clusters based on sink proximity and selects Cluster Heads using parameters like residual energy, neighbor density, and distance to the sink, aided by a spectrum-aware AODV routing scheme. Another recent study presents WEMER (WEdge MERging), a novel approach to mitigate the energy hole problem in wireless sensor networks. Instead of relying on static techniques like mobile sinks or redundant nodes, WEMER divides the network into equiangular wedges and dynamically merges neighboring wedges when their collective residual energy drops below a threshold. An optimized Head Node (HN) selection mechanism further minimizes transmission energy among nodes^16^. In a multi-hop wireless sensor network (WSN), nodes closer to the Base Station (BS) relay a proportionally larger number of packets, resulting in disproportionate energy depletion. Let S denote the set of deployed nodes and b the BS location. The total forwarding (relay) load handled by node i is:

1 $$\begin{aligned} L_i = \sum _{j \in S} f(j \rightarrow i) \end{aligned}$$

As shown in Eq. (1), the relay load of a node *i* depends on its descendants.

2 $$\begin{aligned} & f(j \rightarrow i) = {\left\{ \begin{array}{ll} 1, & \text {if node } i \text { forwards data originating from node } j, \\ 0, & \text {otherwise}. \end{array}\right. } \end{aligned}$$

3 $$\begin{aligned} & D_i = \sum _{j \in S} f(j \rightarrow i) \end{aligned}$$

4 $$\begin{aligned} & E_i = E_{\text {tx}}(p, d_{i,b}) \cdot D_i + \sum _{k \in C_i} E_{\text {rx}}(p) \end{aligned}$$

- $C_i$ denotes the set of child nodes forwarding data through node *i*.
- $E_{\text {tx}}$ and $E_{\text {rx}}$ follow the first-order radio energy model.
- *p* represents the packet size.
- $d_{i,b}$ is the distance between the node *i* and the base station (BS) or relay.

5 $$\begin{aligned} & D_i \gg D_j, \quad \forall j \in S \ \text {such that} \ d_{j,b} > d_{i,b} \end{aligned}$$

6 $$\begin{aligned} & \frac{dE_i}{dt} \gg \frac{dE_j}{dt} \end{aligned}$$

7 $$\begin{aligned} & \exists \, i \in S: E_i(t^*) = 0 \ \wedge \ d_{i,b} = \min _{k \in S} d_{k,b} \end{aligned}$$

This condition implies that nodes closest to the base station deplete their energy first, resulting in network disconnection even though many distant nodes retain unused energy.

This paper introduces a novel routing protocol designed to overcome the limitations found in contemporary routing approaches for WSNs. The central objective of the proposed scheme is to enhance energy conservation among sensor nodes by implementing innovative network partitioning and data forwarding mechanisms. It adopts an unequal clustering strategy that facilitates a balanced distribution of the CH role across the entire network. The maximum cluster size is carefully determined to ensure that no node transmits data beyond the threshold distance defined by the radio energy model. This constraint maintains energy dissipation at a rate proportional to $d^2$, significantly lower than the $d^4$ loss observed in long-distance transmissions. Rather than rotating the CH in every round, the protocol includes an optimized mechanism to adjust the frequency of CH changes, thereby minimizing unnecessary energy expenditure. Integrating mobile data mules alleviates the burden on edge nodes, typically responsible for transmitting large volumes of data over extended distances. As a centralized protocol, it significantly reduces the involvement of sensor nodes in tasks such as cluster formation, CH election, and routing path determination. This reduction in overhead tasks further contributes to energy savings. Data transmission occurs through horizontal level-based paths, effectively addressing the energy hole problem and promoting uniform energy usage throughout the network. Collectively, these design choices lead to substantial energy conservation, extended network lifetime, and improved system stability. By integrating multiple energy-efficient techniques into a single routing solution, the proposed protocol demonstrates superior performance in terms of longevity and stability when compared to existing routing protocols. In grid-partitioned unequal clustering with an MDC that dwells 1s at each grid’s vertical center, multi-hop backbone load is reduced, yet local imbalance persists: CHs and nodes near MDC rendezvous points process more control and aggregation traffic, depleting energy faster. To counter this, we (i) use unequal cluster sizing so high-load cells host smaller clusters, (ii) adopt epoch-based CH tenure with residual-energy thresholds to avoid per-round re-elections yet prevent CH starvation, and (iii) publish a coarse MDC tour so CHs batch transmissions and suppress frequent re-scheduling beacons. These choices improve energy balance without adding significant signaling.

The remainder of this paper is structured as follows: “Related works” reviews the existing literature relevant to this study. “System model” details the system model, including the assumptions made for the network architecture, radio energy consumption, and criteria for determining cluster sizes. “Proposed solution” provides a thorough description of the proposed routing protocol. “Simulation results” presents the simulation setup, experimental results, and performance evaluation. Finally, “Conclusions and future works” offers concluding remarks and highlights potential directions for future research.

## Related works

LEACH (Low Energy Adaptive Clustering Hierarchy) was the first hierarchical routing protocol based on a distributed clustering mechanism^5^. It functions on a round-based system wherein each node generates a random number between 0 and 1 at the start of each round. A node becomes a cluster head (CH) for that round if its random number is below a predefined threshold, after which it broadcasts a CH announcement to nearby nodes. Although LEACH introduced notable energy-saving techniques, it suffers from several limitations: (i) CH selection does not consider node energy levels, (ii) CHs communicate with the BS through direct single-hop transmissions, and (iii) CHs are randomly located, alleviating the burden on nearby charge carrier formations. To address these drawbacks, various enhancements have been proposed in the literature^8^. One such enhancement is LEACH-C (LEACH-Centralized), introduced by Heinzelman et al., where CH selection and cluster formation are managed centrally by the BS, and the number of CHs is fixed across all rounds^17^. This centralization reduces the overhead of forming new clusters repeatedly. Despite improved performance over LEACH, LEACH-C still faces issues such as reliance on single-hop CH-to-BS communication and suboptimal CH selection. Younis et al. developed the HEED (Hybrid Energy-Efficient Distributed Clustering) protocol, which improves CH selection by considering both residual energy and intra-cluster communication cost. HEED reduces energy consumption by avoiding frequent CH elections. However, its multi-level clustering architecture is still prone to the energy hole problem. To address energy dissipation during data transmission, MS-LEACH was proposed, which limits energy loss by enforcing transmission distances that yield quadratic, rather than quartic, energy decay^18^. It allows for both single-hop and multi-hop intra-cluster communication. For large clusters, a shortest-path tree structure is employed for efficient data routing within the cluster. Multi-hop LEACH extends the basic LEACH protocol by enabling CHs to forward data to the BS via multiple intermediate CHs^19^. While this multi-hop strategy improves energy efficiency, it also intensifies the energy hole problem due to increased traffic near the BS. EEM-LEACH introduces an alternate approach where nodes close to the BS bypass clustering and transmit directly to it^20^. This design choice mitigates the energy hole effect by alleviating the burden on nearby charge carriers. In this protocol, route discovery is based on minimizing communication cost. The energy hole problem persists across most multi-hop routing protocols and arises from the disproportionate forwarding load placed on nodes situated near the BS. Our proposed scheme addresses this issue more effectively through a combination of unequal clustering and mobile data collection.

Lui et al. proposed a grid-based approach called LPGCRA to address the energy hole problem^21^. In this protocol, the transmission distance within clusters is the key factor for selecting CHs. The chosen CHs also act as relays, with their count being limited to balance energy usage across the network. This method ensures that clusters closer to the BS consume less energy. Although LPGCRA performs effectively in small-scale WSN deployments, it struggles to maintain efficiency in larger network areas. Similarly, the grid-based routing protocol DBSCAN selects the CH within each cluster as the node closest to the BS^22^. However, this choice leads to increased intra-cluster communication costs and subsequently reduces the network lifetime. Jannu and Jana introduced the Low Power Grid-Based Cluster Routing Algorithm (FBECS)^23^, which also employs a grid-based network division strategy primarily designed to mitigate energy holes. Their scheme divides the network into equal-sized grids and selects the node with the highest residual energy as the CH in each grid. Despite its merits, FBECS relies on single-hop communication from CHs directly to the BS, limiting its applicability in extensive WSN deployments. Gupta and Pandey proposed an enhanced distributed unequal clustering protocol named EADUC, which is energy-aware and designed to alleviate hot spot issues through multi-hop communication^10^. This protocol forms unequal clusters based on the BS’s location and the node’s remaining energy. CH selection incorporates node degree alongside these factors. Relay nodes for multi-hop transmission are selected based on their distance to CHs and energy costs. Nevertheless, the protocol’s reliance on heterogeneous node capabilities increases overall system complexity and cost.

Shah et al. presented a three-tier reference architecture in which the lowest layer consists of numerous sparsely placed sensor nodes^24^. The intermediate layer features mobile data carriers, called MULES, that randomly roam the network to collect data from sensor nodes. These MULES subsequently deliver the gathered data to an upper-layer access point, which is IP-enabled and connected to a broader network infrastructure. This setup behaves similarly to an opportunistic network characterized by unpredictable MULE mobility and increased data transmission delays. Singh et al. developed a grid-based clustering routing protocol integrated with mobile mules^25^. Their design uses an odd-even level routing strategy for forwarding data within each level, while mobile mules collect data from the boundary CHs of those levels. Although the scheme deploys two mules, only one visits a level per round to limit parallelism. Due to its centralized clustering methodology, this approach may face scalability challenges in large-scale networks. Kaswan et al. proposed two novel data collection schemes, EDT and DAEDT, which leverage the controlled mobility of mobile sinks. These protocols calculate rendezvous points (RPs) based on energy density metrics in a flat network where communication is restricted to single-hop transmissions^26^. EDT identifies RPs by evaluating the impact of each sensor node on its neighbors within communication range and selects those with the lowest impact. It then constructs a Traveling Salesman Problem (TSP) route to cover all selected RPs. However, EDT does not consider application-specific delay constraints, making it unsuitable for latency-sensitive applications, and has a worst-case time complexity of $O(n^3)$ for n nodes. The DAEDT algorithm extends EDT by imposing a user-defined delay bound on the TSP route, which reduces the number of RPs but increases communication distances. This relaxation results in sensors occasionally communicating beyond a one-hop range, increasing energy consumption. Singh et al.^25^ further enhanced their previous work by incorporating unequal clustering and diverse mobile mule trajectories^27^, introducing a mechanism to calculate near-optimal CH change and cluster size reduction factors.

**Fig. 1:**
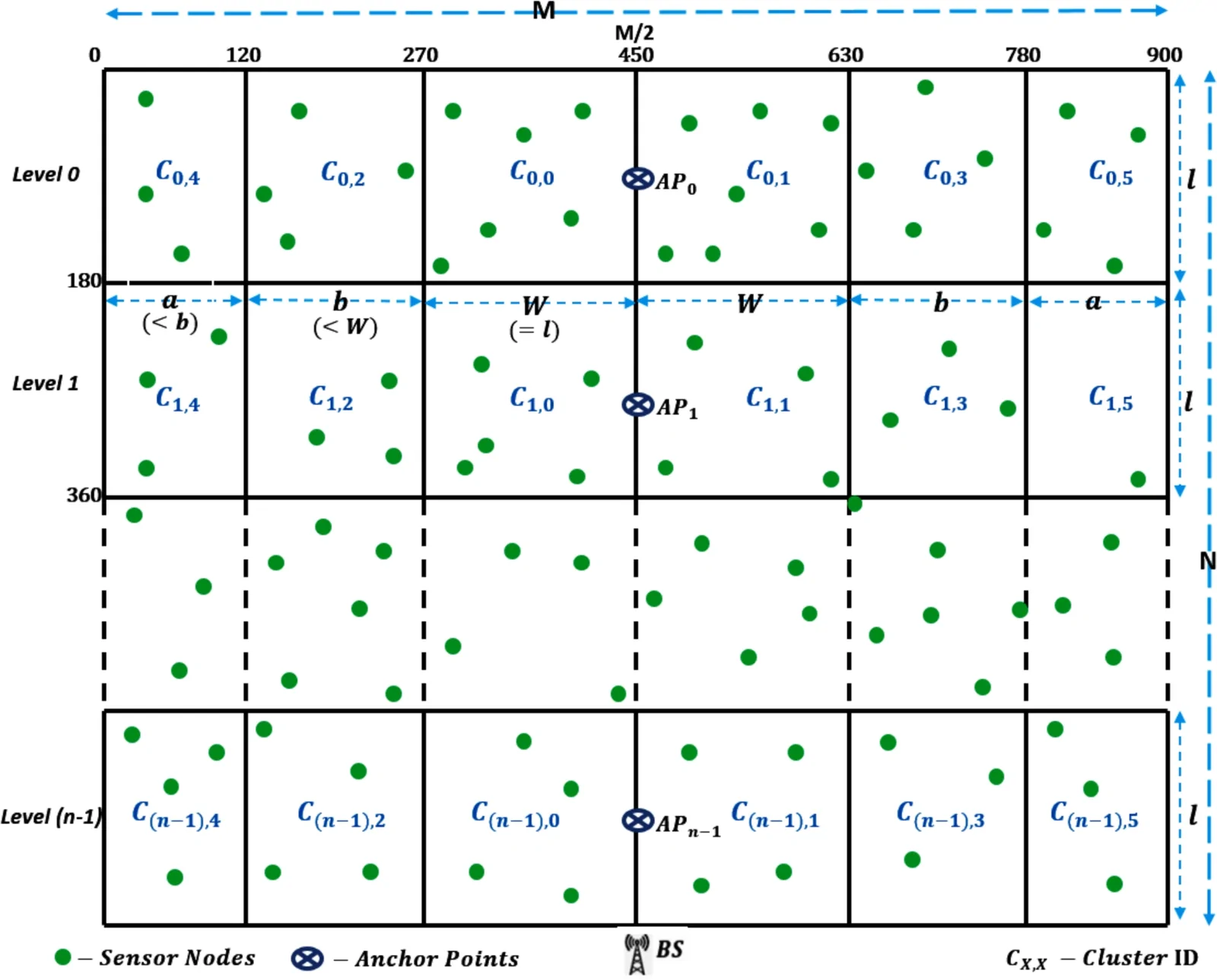
Division of network into unequal clusters.

A scheme to minimize energy holes using a dual-sink approach with dynamic load transition for data transmission is introduced in^28^. The two sinks cooperate by shifting the communication load toward areas with lower energy consumption, thereby balancing energy usage across the network. While this method demonstrates improved energy efficiency, its main drawback lies in the increased complexity of the algorithm. Dogra et al. proposed a dynamic clustering protocol designed to mitigate energy holes in WSNs^29^. The sensing field is divided into two distinct regions, each managed by a gateway node responsible for collecting data from all nodes within its region. Clustering occurs independently in each region, with two cluster heads (CHs) per cluster. At any given time, only one CH remains active, while the other is placed in sleep mode to conserve energy. Although this technique extends the network lifetime, it introduces additional overhead due to multiple CH and gateway selections, reducing its effectiveness in large-scale networks. Jianpo et al. presented an Energy Hole Suppression Routing Algorithm (EHSRA) that fixes the transmission sequence of CHs^30^. The protocol monitors the energy hole status in each round and adjusts data transmission accordingly to mitigate energy depletion. This approach works well for small networks but may not scale effectively for larger deployment areas. Another recent approach, a load-balancing clustering algorithm (LBCF) detailed in^31^, selects CHs through a fuzzy logic system that evaluates residual energy and distance to the base station. The load on each sensor is computed based on the total number of transmissions it handles, reflecting its load capacity. The authors claim that this method reduces energy holes for both equal and unequal clustering schemes. However, the CH selection criteria are suboptimal, limiting overall performance. An Energy-Aware Method (EAM) for chain-based routing in wireless sensor networks (WSNs), focusing on reducing energy consumption and enhancing network lifetime, was proposed by Vahabi et al^32^. EAM employs a greedy algorithm to transmit data between layers, considering both node energy and distance to the sink. It significantly reduces end-to-end delay and improves efficiency. Simulation results show that EAM decreases energy usage by at least 41.71% and extends network lifetime by 58.28% compared to existing methods. Vahabi et al. introduce a novel deep learning-based movement path for mobile sinks in wireless sensor networks (WSNs), termed Reinforcement Learning Movement Path (RLMP)^33^. Unlike traditional random, controlled, or predefined paths, RLMP enables mobile sinks to dynamically target high-data regions, improving routing performance. The approach enhances end-to-end delay, energy efficiency, delivery ratio, and network lifetime. Simulations using NS-3 show RLMP outperforms existing methods, extending network lifetime by at least 32.48% (Fig. 1).

The comparison of the existing works explaining their main contribution, advantages, and disadvantages is summarized in Table 1.

**Table 1:** Existing works with their benefits and drawbacks.

Author names	Main contribution	Advantages	Limitations
Liu et al.^21^	A grid-based scheme for reducing the energy hole issue named LPGCRA. CH works as the relay node for forwarding the data	Balanced energy consumption and the nearest clusters of the BS consume less energy	CH selection is not optimal; it can be improved
Jannu and Jana^23^	Grid-based clustering and routing algorithms, collectively referred to as GFTCRA, are distributed by nature and include runtime fault detection and maintenance for faulty sensor nodes or cluster heads (CHs)	Effective for energy efficiency, load balancing, and fault tolerance together	For large-area WSNs, overheads will be more
Gupta and Pandey^10^	Energy-aware distributed unequal clustering, known as EADUC, maintains the same clusters for several rounds by effectively reducing clustering overhead	Network lifetime is improved significantly	Heterogeneity, which amplifies the overall cost and complexity
Nitesh et al.^26^	Dynamic path planning for mobile sink with delay bound, rendezvous point (RP) selected based on nodes density	Network lifetime increases compared with communication range and the number of nodes	Transmission overheads for path planning and data collection at RP
Singh and Kumar^27^	Data collection using two mobile sinks with three different speeds in a rectangular sensing area	Low delay with maximum data collection	Maintaining synchronization between edge CHs and mobile sinks is a challenging task
Chen et al.^28^	Two sinks work cooperatively and adjust energy consumption by shifting the load towards a less energy-consuming area.	Showing a better performance in terms of energy efficiency	High complexity and overheads
Dogra et al.^29^	The sensing area is divided into two halves, and a gateway node is selected in each half for collecting data from all the CHs of that half	Transmission is efficiently done using multiple CHs and gateway nodes	Due to multiple CHs and gateways, the extra overhead is high
Jianpo et al.^30^	Fixing the transmission order of all the CHs to forward their data to sink node	Reduced routing overhead, and low delay	Not suitable for large area WSNs
Edla et.al^31^	To introduce an innovative algorithm that accounts for both the relative velocity of vehicle nodes and intra-cluster distances while ensuring link reliability	An enhanced link reliability and a traffic-aware, low-latency schema (TaLAR) are proposed	Reduced performance in dynamic networks caused by routing overhead

## System model

### Network model

The wireless sensor network (WSN) is deployed over a *M × N* rectangular region, where *S* homogeneous sensor nodes are uniformly distributed. After deployment, all nodes remain static and are aware of their geographical coordinates. The base station (BS) possesses global knowledge of all nodes, including their node IDs, positions, and remaining energy levels.

8 $$\begin{aligned} D_0 = \sqrt{\frac{\varepsilon _{fs}}{\varepsilon _{mp}}} \end{aligned}$$

A Mobile Data Collector (MDC) moves along a deterministic vertical path through the center of the network field. The MDC pauses at predefined Anchor Points (APs), with one AP at each network level.

9 $$\begin{aligned} 0 < v \le v_{\max } \end{aligned}$$

The MDC is assumed to have unlimited energy.

10 $$\begin{aligned} & t_{\text {wait}} = \text {stop time at each anchor point} \end{aligned}$$

11 $$\begin{aligned} & T_{\text {sweep}} = \frac{N}{v} + m \cdot t_{\text {wait}} \end{aligned}$$

- Sensor nodes are randomly deployed.
- All nodes are initialized with equal energy $E_0$.
- Unequal grid regions are formed based on derived width constraints to balance energy consumption.

### Energy model

Our protocol uses the same radio energy model as described in^5^. Equations (12) and (13) provide details of energy consumption during transmission and reception of messages by sensor nodes. The notations used in the equations are mentioned in Table 2. The energy consumed in the transmission of *p* a bit packet of data over a *d* is given by Eq. (12).

12 $$\begin{aligned} \begin{aligned} E_{trans}({p},d)= {\left\{ \begin{array}{ll} E_{e}*{p}+e_{fsf}*{p}*(d)^2, & d < D_0 \\ E_{e}*{p}+e_{mpf}*{p}*(d)^4 , & otherwise \end{array}\right. } \end{aligned} \end{aligned}$$

The dissipation of energy during the reception of a message of *l* bit packets is derived by using Eq. (13).

13 $$\begin{aligned} E_{recv}({p})=E_{e}*{p} \end{aligned}$$

The threshold radio range ($D_0$) is calculated with the help of Eq. (14).

14 $$\begin{aligned} D_0=\sqrt{\frac{e_{fsf}}{e_{mpf}}} \end{aligned}$$

### Cluster size selection

To ensure reliable intra-grid communication, the maximum transmission distance within a grid is constrained to remain below the threshold distance $D_0$. The maximum distance between any two nodes within a grid is given by

15 $$\begin{aligned} d_{\max } = \sqrt{l^{2} + (2w)^{2}}, \end{aligned}$$

where *l* denotes the grid length and *w* represents the grid width.

To guarantee free-space propagation, the following condition is enforced:

16 $$\begin{aligned} d_{\max } \le D_0. \end{aligned}$$

Substituting (15) into (16) and solving for *w* yields

17 $$\begin{aligned} w \le \frac{D_0}{\sqrt{5}}. \end{aligned}$$

This constraint ensures that all communication links operate within the free space propagation region characterized by a $d^{2}$ path-loss model, thereby avoiding the energy-intensive multipath fading region governed by a $d^{4}$ loss model. Extensive simulation results further confirm that the minimum energy variance across sensor nodes is achieved when the grid width is set to *w*.

**Fig. 2:**
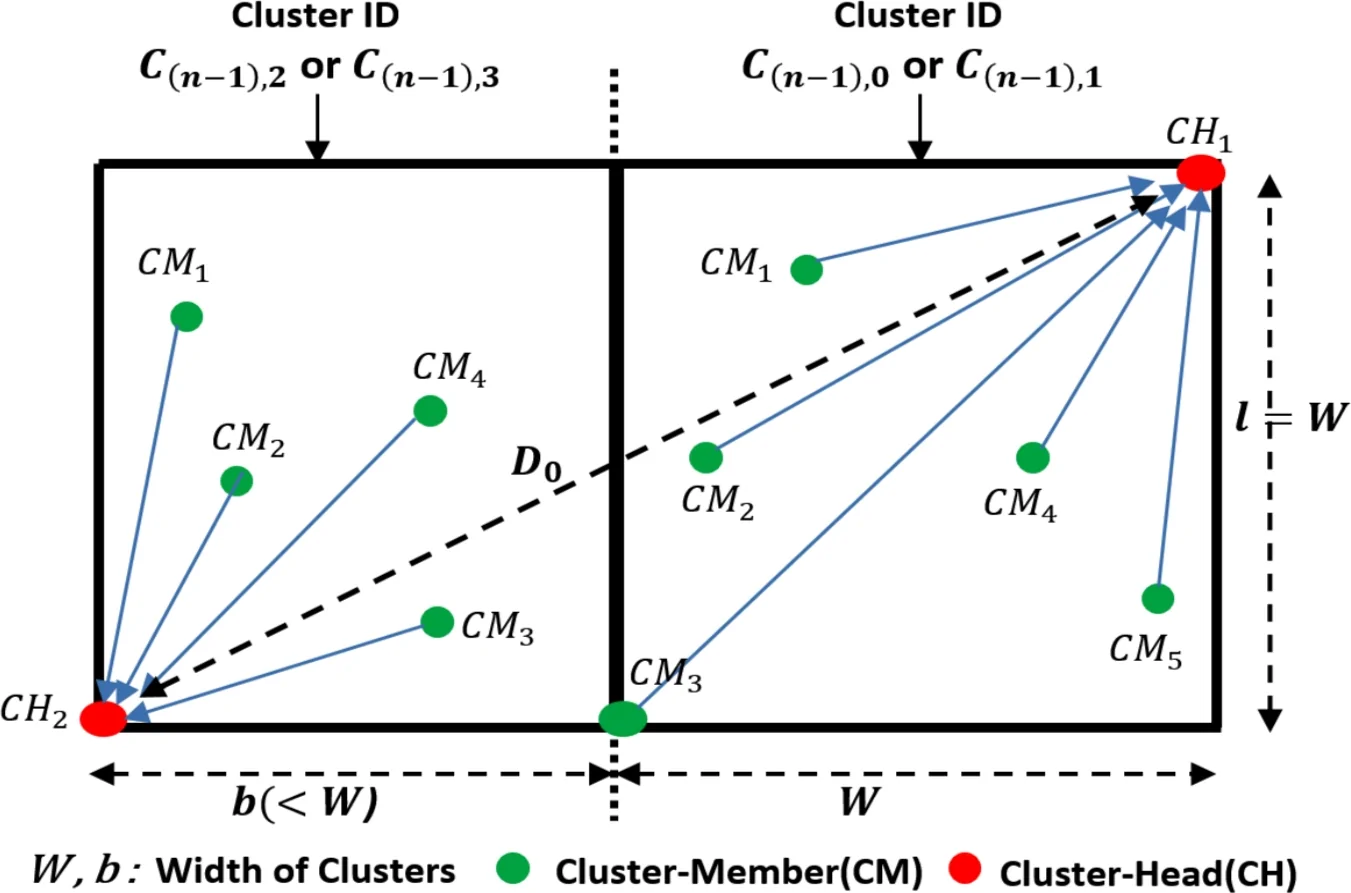
Communication between two clusters.

Figure 2 is considered for the calculation of the width of the largest cluster $D_o$ such that the inter-cluster communication distance between two sensor nodes of adjacent clusters never exceeds $D_o$. As shown in Fig. 2, the two cluster head sensor nodes, namely CH$_1$ and CH$_2$ are at diagonally opposite corners of the rectangle formed by them together. For determining the maximum possible diagonal communication distance between them, suppose the two adjacent clusters C$_{{level}_{id},(numCL-1)}$ and C$_{{level}_{id},(numCL-3)}$ are of the same size; then

Width of cluster C$_{{level}_{id},(numCL-3)}$, *w* = *b* = *W*

From Eq. (17), it is clear that if the maximum width of the cluster is ${D_0}/\sqrt{5}$ then the maximum transmission distance for any node is equal to the threshold distance $D_0$ for any possible inter-cluster communication within the network, as this distance is the diagonal distance between two adjacent clusters, as shown in Fig. 2.

In a real scenario, *b*    <    *W*.

### Overhead analysis

#### Communication overhead

The communication overhead of the proposed scheme comprises multiple components. Intra-cluster communication is scheduled using TDMA, which incurs a complexity of

$$\mathcal {O}(N_c)$$,

where $N_c$ denotes the number of nodes within a cluster. The inter-cluster forwarding among cluster heads (CHs) follows a chain-based routing structure and incurs a complexity of

$$\mathcal {O}(C_H)$$,

Where $C_H$ is the total number of CHs. The communication between the Mobile Data Collector (MDC) and CHs at anchor points is constant, i.e.,

$$\mathcal {O}(1)$$,

Only aggregated data packets are exchanged. The overhead due to CH rotation control messages is minimal and considered negligible compared to data transmission traffic.

#### Computation overhead

The computational overhead primarily arises from cluster head selection and network maintenance operations. The CH selection process within each cluster has a computational complexity of

$$\mathcal {O}(n_c)$$.

The threshold-based CH update mechanism executed by the base station incurs a complexity of

$$\mathcal {O}(C)$$.

In addition, MDC path scheduling involves a constant-time operation, resulting in

$$\mathcal {O}(1)$$,

computational complexity.

Overall, the incorporation of the MDC significantly reduces backbone relay traffic, achieving an approximate reduction of *40%*–*55%* compared to static sink-based approaches.

**Fig. 3:**
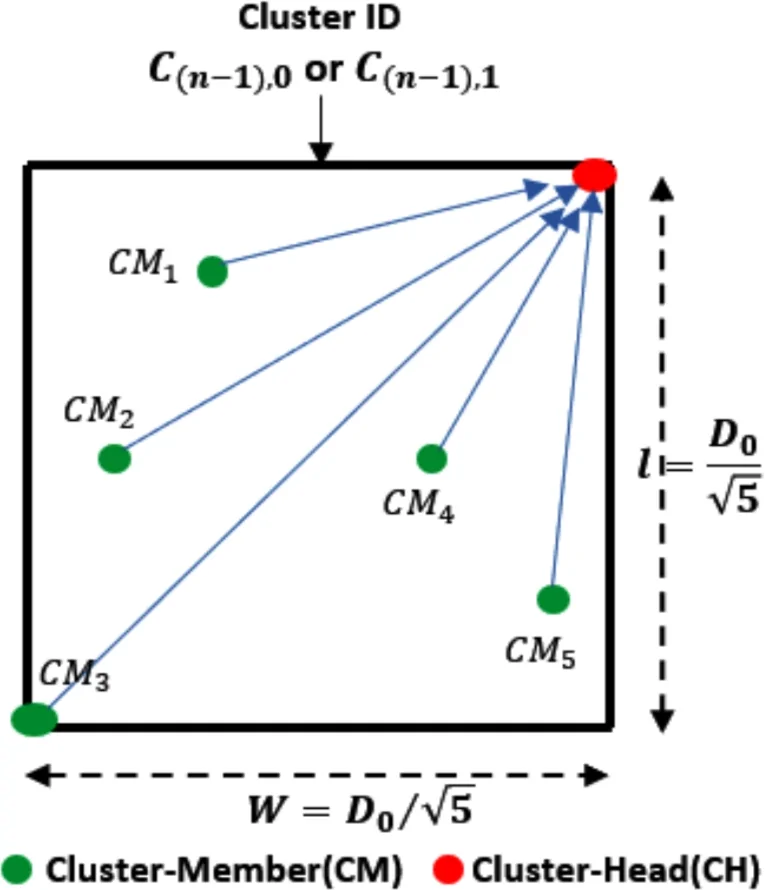
Communication within cluster.

Therefore, the maximum transmission distance for any node is always less than the threshold transmission distance $D_0$ for a maximum cluster width ${D_0}/\sqrt{5}$ for inter-cluster communication. Based on the first-order radio model, we have calculated the maximum communication range, denoted as D$_0$, which represents the diagonal distance across a rectangular grid. Cluster members can cover this distance from any corner of the grid. To optimize coverage, mobile data collectors (MDCs) are deployed to move along the center and both sides of the network area. We apply the same type of unequal clustering strategy across the network. By doing so, we effectively reverse the unequal distribution of clusters typically caused by distance constraints.

To ensure that the maximum transmission distance for any node is always less than the threshold transmission distance $D_0$ for any communication in the entire network, we must verify this for intra-cluster communication, given the calculated maximum cluster width *W*. Figure 3 shows communication between CM and CH of the largest cluster in the worst-case scenario when they are at diagonally opposite corners of the grid.

In this case, the distance between the two most distant nodes within the cluster is also less than $D_0$. The maximum cluster width *W* ensures that the communication distance between any two nodes within the cluster is always less than $D_0$. Hence, the maximum calculated width *W* of the cluster ensures that all the nodes in the network deplete their energy at a rate of $d^2$, which is, respectively, much less than $d^4$.

**Table 2:** Notations used in our scheme.

Symbols	Descriptions
M and N	Rectangular area of interest
$D_0$	Threshold distance
S	Numbers of sensors
CM	Cluster member
MN	Member node
*W*	Maximum width of the grid or cluster in the network
*l*	Width of a level or Length of the cluster
L	Total number of levels
r	Cluster width decreasing rate
*CHC*	Multiplier having integral value between 1 to 10
S$_i$	Sensor node ID
level$_{id}$	Level ID
C$_{{level}_{id},i}$	ID of the cluster belongs to level$_{id}$
CH$_{{level}_{id},i}$	CH belongs to cluster C$_{{level}_{id},i}$
C$_{{level}_{id},i}$[ ]	Node IDs of cluster C$_{{level}_{id},i}$
numSC$_{{level}_{id},i}$	Total number of nodes in cluster C$_{{level}_{id},i}$
numCL	Total clusters in a level
E(Th)$_{{level}_{id},i}$	Threshold energy of a node to become CH
nodeSet[ ]	Node IDs whose energy is E > E$_{avg}$
dist[ ]	Distance of nodes from the centroid in a cluster
E$_{min}$	Active node’s threshold energy
*p*	Packet size transmitted by sensor node
E$_{recv}({p})$	Energy consumes to receive *p*
E$_{trans}({p},d)$	Energy consumes to transmit a *p* to a distance dist$_{trans}$
E$_{e}$	Energy consumes by electronic circuit
e$_{fsf}$	Amplification energy in free space model
e$_{mpf}$	Amplification energy in multi-path fading model
AP$_{{level}_{id}}$	Anchor point at each level$_{id}$

## Proposed solution

The proposed protocol is a centralized protocol based on a fixed clustering approach. The protocol consists of four stages: (i) Cluster Formation, (ii) Setup, (iii) Data Forwarding, and (iv) Steady. The proposed scheme employs a chain-based routing structure inspired by the PEGASIS protocol^34^. In this approach, sensor nodes located within the same grid row are connected sequentially to form a communication chain. Each grid CH transmits its aggregated data to the nearest neighboring CH, and the final CH in the chain forwards the combined data to the MDC. This hierarchical routing minimizes long-distance transmissions, balances energy usage among grid nodes, and significantly enhances the overall network lifetime and energy efficiency. The detailed description of all the proposed algorithms is discussed below.

### Algorithm A: grid formation

Input: Node coordinates, network dimensions, $D_0$

Output: Level boundaries, grid identifiers The base station computes network levels and forms unequal grids such that the grid width satisfies the constraint *w*. Each node is assigned a grid identifier based on its location. Time complexity: $\mathcal {O}(S)$

### Algorithm B: cluster head (CH) selection

Input: Nodes within a grid, residual energies

Output: Cluster Head identifier Nodes with residual energy greater than the average cluster energy are selected as CH candidates. The node closest to the grid centroid is elected as the CH. Time complexity: $\mathcal {O}(n_c)$

### Algorithm C: CH change decision

Input: Current CH energy $E_{\text {CH}}$

Output: CH retain or reselect decision The CH is retained if

18 $$\begin{aligned} E_{\text {CH}} \ge \text {CHC} \cdot E_{\text {th}}, \end{aligned}$$

Otherwise, a new CH is reselected for the grid. Time complexity: $\mathcal {O}(1)$

### Algorithm D: routing path discovery

Input: Cluster Heads of all grids

Output: CH-to-CH forwarding chain A routing chain is established among neighboring CHs toward the MDC path to enable multi-hop data forwarding. Time complexity: $\mathcal {O}(C)$

### Algorithm E: MDC data collection

Input: Anchor Point positions, routing chain

Output: Aggregated sensed data The MDC pauses at each anchor point and collects aggregated data from the corresponding CH via the established routing chain. Time complexity: $\mathcal {O}(L)$

Table 2 illustrates the notations used in the proposed protocol.

**Algorithm 1:**
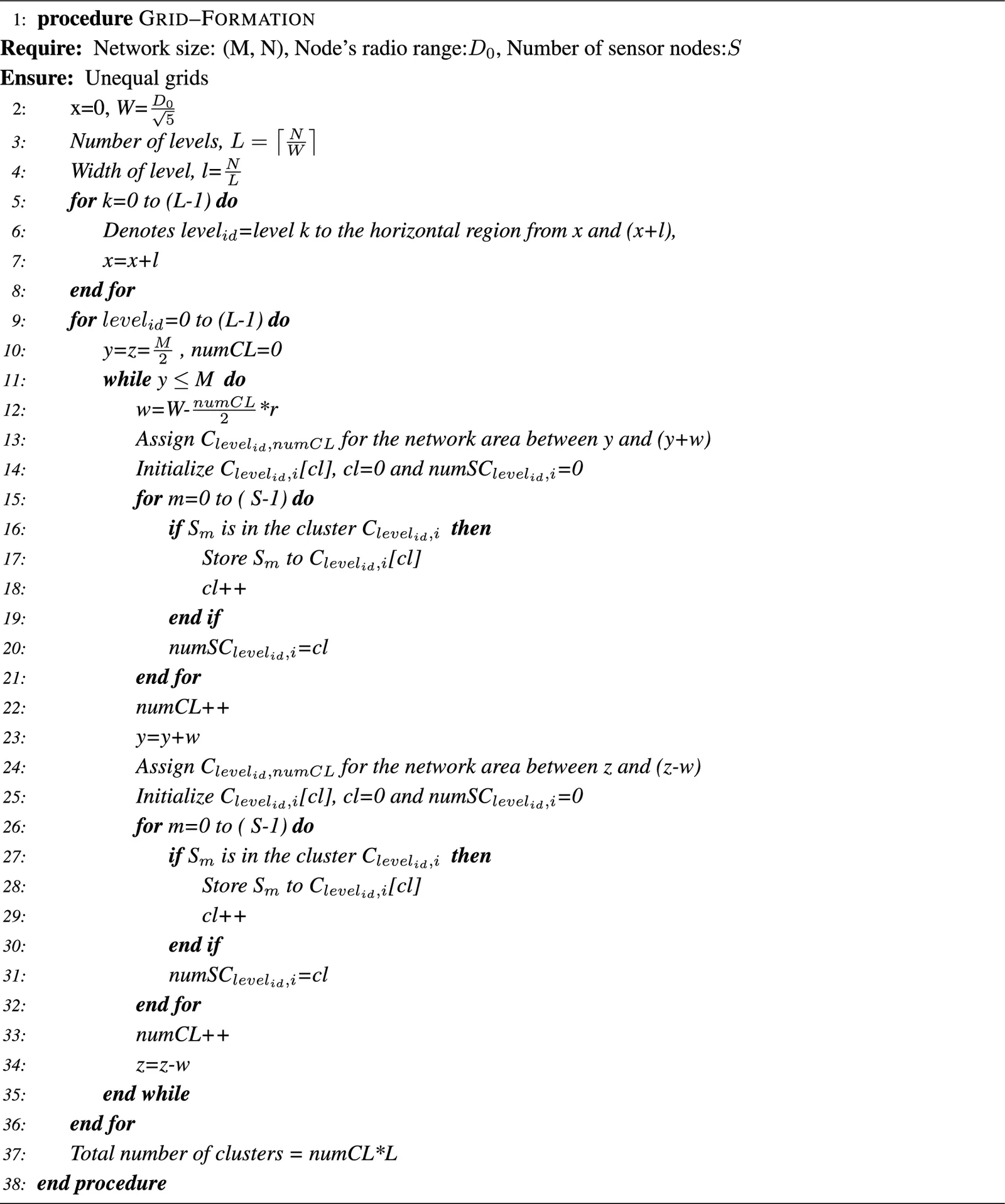
Unequal grid formation algorithm.

The proposed MDC-based unequal grid clustering scheme operates in a round-based manner through a structured sequence of phases. Initially, the base station (BS) partitions the sensing field into unequal grids by computing network levels and grid widths while enforcing the constraint $w \le \frac{D_0}{\sqrt{5}}$ to ensure reliable intra-grid communication. For each grid, the BS determines the centroid and selects cluster head (CH) candidates with residual energy higher than the network average; the node closest to the centroid is elected as the CH to minimize communication overhead. Subsequently, a deterministic vertical path is assigned to the Mobile Data Collector (MDC), and anchor points are established at grid centroids. During the setup phase, a TDMA schedule is created for intra-cluster communication, and a CH-to-CH routing chain is formed toward the MDC path. In the data transmission phase, cluster members transmit data to their respective CHs using TDMA, CHs aggregate and forward the data along the routing chain, and the final CH delivers the aggregated data to the MDC at the corresponding anchor point. The MDC pauses at each anchor point to collect data, thereby alleviating energy depletion near the base station. After each round, the BS evaluates the residual energy of CHs and triggers CH reselection if the energy falls below a predefined threshold; network parameters are then updated, and the process repeats until termination.

### Cluster formation phase

The prime objective of this phase is the division of the network into levels and grids (clusters) of varying sizes with unique IDs. Algorithm 1 elucidates the complete process of this stage.

**Algorithm 2:**
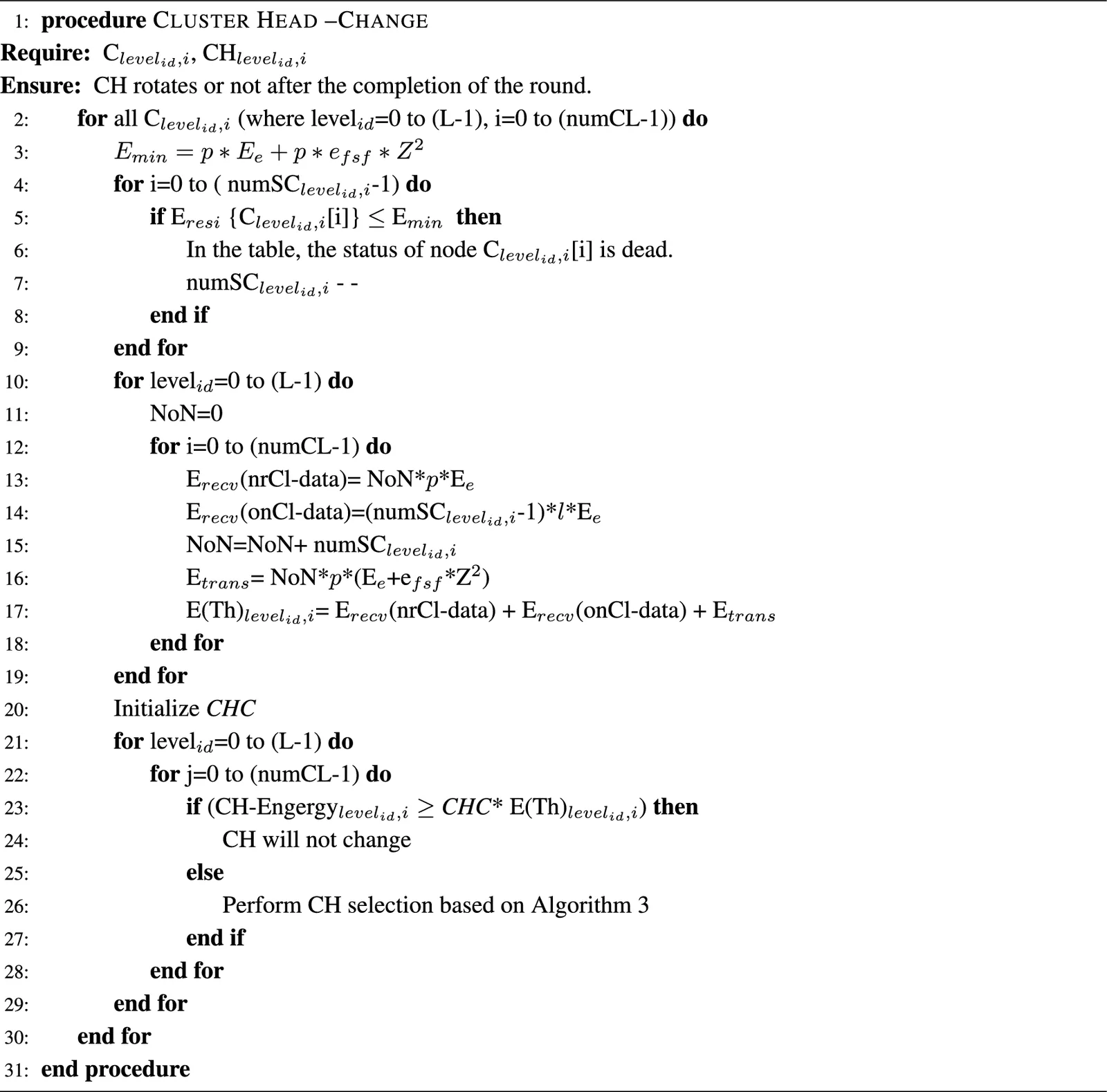
CH change algorithm.

Algorithm 1 is executed only once at the start of the network setup phase by the BS. It calculates $D_o$ for the network and divides the network into levels, denoted as level$_{id}$. Then, it divides each level into unequal-sized grids, called clusters. It assigns a unique ID, C$_{level_id}$, to each cluster and assigns sensor nodes to that cluster. It maintains a record of cluster IDs and node IDs belonging to that cluster.

A vertical path is defined through the center of the network to guide the movement of the Mobile Data Collector (MDC). Along this path, specific anchor points (APs) are positioned where the MDC pauses to gather data from the Cluster Head (CH) nodes. The cluster size decreases linearly with a constant rate *r* as the level moves farther from these APs, ensuring that clusters near APs—responsible for higher data forwarding loads—remain smaller and more energy-efficient. Larger clusters located farther away distribute the CH selection load among more nodes, reducing the frequency of CH re-selection and conserving node energy. This adaptive design effectively prevents the formation of energy holes and extends network lifetime. Figure 1 illustrates this process, showing the division of the network into equal-width levels and unequal-sized clusters and the assignment of unique level and cluster IDs after executing Algorithm 1.

### Setup phase

The setup phase includes Algorithm 2 and Algorithm 3, namely the CH Change Algorithm and the CH Selection Algorithm. Both algorithms are executed by the BS, outlining the working of this phase. Algorithm 2 determines whether the current CH of a cluster will change or continue acting as CH after the completion of a round. At the start of each round, it identifies the nodes within each cluster that are unable to transmit data due to lower energy levels and sets their status as ‘dead’ nodes in its record table. After that, it calculates the threshold energy for each cluster and compares it to the energy of their current CHs. If the energy of the current CH is greater than *CHC*(an integral value) times the calculated threshold energy of that cluster, CH will not change. Otherwise, Algorithm 3 is executed for the selection of a new CH among the cluster’s alive nodes. *CHC* is a predefined integral value that affects the frequency of rotation of CH in a network. Algorithm 3 calculates the centroid and average energy of the active nodes for that cluster. The node nearest to the centroid and having greater energy than the average energy of the cluster is selected as the new CH. The combination of both algorithms optimizes the frequency of CH rotation in the network. This reduces the overhead energy loss of the network for changing CH in each round.

### Data forwarding phase

In this phase, data forwarding routes are discovered to transmit the data packets towards APs. In each round, the BS performs this task.

Initially, it selects the CH$_{{level}_{id},i}$ of the cluster C$_{{level}_{id},i}$. It searches the CH$_{{level}_{id},i-2}$ of the apparent neighbor cluster C$_{{level}_{id},i-2}$ toward the AP$_{{level}_{id}}$. If C$_{{level}_{id},i-2}$ is present in the same level, then the CH of that cluster works as a relay node for CH$_{{level}_{id},i}$. If one-hop neighbor CH is not found at the same level, then it searches the upper-level CH$_{{level}_{id}+1,i-2}$ and, lastly, the lower-level CH$_{{level}_{id}-1,i-2}$. Each CH performs this process until all the CHs have received their relay node for data forwarding. The process can be easily analyzed by using a network division diagram, as shown in Fig. 1. Algorithm 4 illustrates the route discovery for data forwarding in this phase. The total energy consumed in transmission and reception of any packet $(E_{TxRx})$ in a cluster can be calculated by Eq. (21) using Eqs. (19) and (20) where $data_{onCl}$ and $data_{nrCl}$ are the total data of one cluster and its neighbor’s cluster total data, respectively.

**Algorithm 3:**
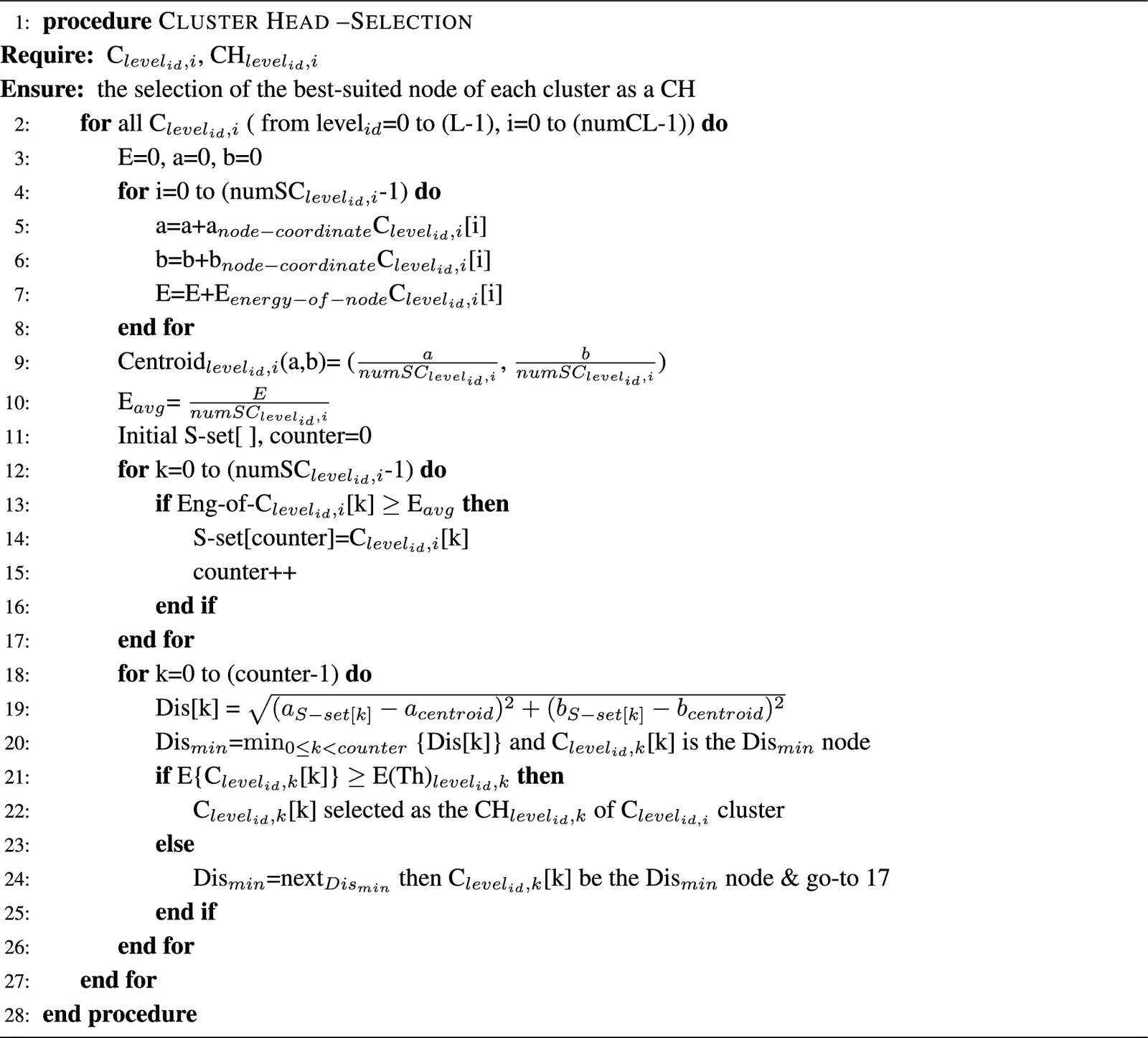
CH selection algorithm.

19 $$\begin{aligned} & \begin{aligned} E_{trans}=(data_{onCl} + data_{nrCl})*E_{e} + (data_{onCl} + data_{nrCl} )*e_{fsf} *D_0^2 \end{aligned} \end{aligned}$$

20 $$\begin{aligned} & E_{recv}=(data_{onCl}+data_{nrCl})*E_{e} \end{aligned}$$

21 $$\begin{aligned} & E_{TxRx}= E_{trans} + E_{recv} \end{aligned}$$

**Algorithm 4:**
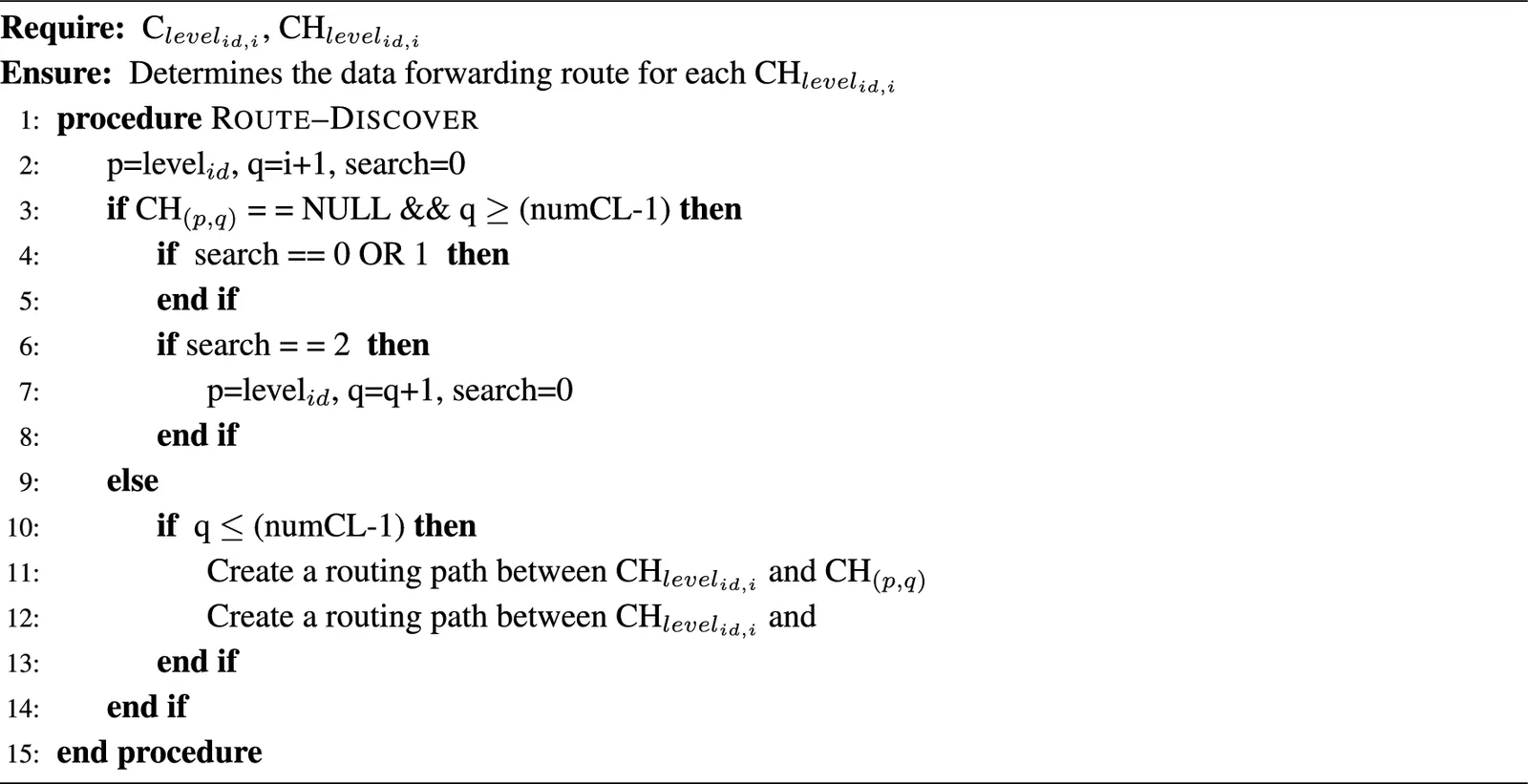
Route discovery algorithm.

### Steady phase

The TDMA time-slot allocation to CMs, CHs, and MDC for transmission and reception of the sensed data is the key task of this phase. Due to the random distribution of nodes in the network, each cluster has a different number of nodes. Therefore, the BS detects the maximum number of node clusters (max_numSC$_{{level}_{id},i}$) and assigns the maximum TDMA time slots to that cluster. Suppose the BS allots *t* seconds to each MN in their TDMA slots; then the maximum TDMA time slots will be $t*(max\_numSC_{{level}_{id},i})$ seconds.

For example, if a cluster has 12 nodes, its TDMA time slots will be 11*t Sec. The communication within a cluster is known as intra-cluster communication, and communication between two CHs or relay nodes is called inter-cluster communication. The average one-hop inter-cluster time is $t_{CH}$. The MDS moves with a uniform speed of *v* m/s, and it stops for $t_{wait}$ seconds at each AP to collect data from borderline CHs. Therefore, we can find out the average period $t_{round}$ for a complete round by using Equation 22.

22 $$\begin{aligned} t_{\text {round}} = t_{\text {setup}} + t \cdot (\text {max}\_\text {numSC}_{{\text {level}_{id},i}} - 1) + (t_{\text {CH}} \cdot \text {numCL} / 2) + \frac{N}{v} + t_{\text {wait}} \cdot m \end{aligned}$$

Here $t_{setup}$ is the average time needed to complete the setup phase. After executing the rectangular grid formation phase, setup phase, and data forwarding phase of the first round, the BS knows the total time required for completion of a round with the help of Eq. (22). It also maintains a table that consists of all cluster and CH information. The BS shares this table with all nodes at the beginning of each round. After the lapse of $t_{setup}$ seconds, CMs transmit their sensed data as per their TDMA slots. In each round, nodes wait for control packets from the BS during their $t_{setup}$ time. If sensor nodes receive any control packets, they update their tables and use the updated tables for future communications.

In each round, the BS checks the residual energy of each CH, and if any CH’s residual energy goes below the threshold energy *E*(*Th*), the BS changes and sends a control message for CH change in that cluster. The new CH information is also attached to this control message. It can be calculated *E*(*Th*) using Eq. (23). Here, *CHC* is the CH change factor, which we have fixed at 5 based on the simulation results. The value of *CHC* is 5, which means that CH does not change for 5 rounds. It will change after the 5th round. We have simulated our scheme for *CHC* values ranging from 1 to 10, using 1200 nodes, and analyzed the number of dead nodes in different rounds. For a value of *CHC* = 5, we get the best results, as the number of dead nodes is less in a different range of rounds at this value. Hence, we have selected this value for our simulation. This $E_{TxRx}$ is the combined energy required for transmission and reception of data by a node as calculated in Eq. (21). More frequent CH changes lead to greater energy loss in the network during the transmission and reception of control packets, whereas less frequent changes result in the early death of sensor nodes. Therefore, an efficient CH rotation is needed to prolong the network lifetime.

23 $$\begin{aligned} E_{Th} = \textit{CHC}* E_{TxRx} \end{aligned}$$

**Fig. 4:**
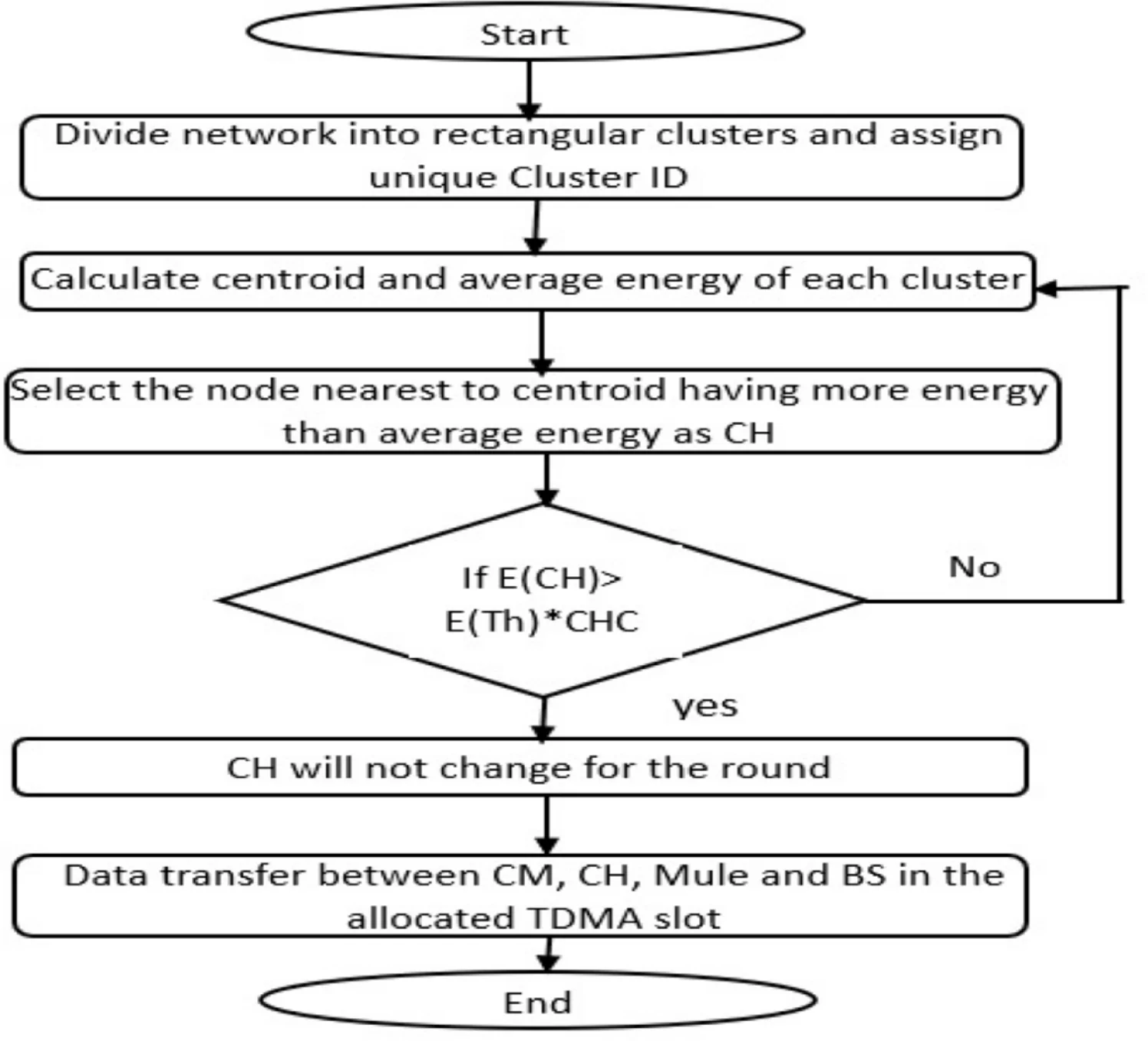
Operational workflow of the proposed protocol.

**Fig. 5:**
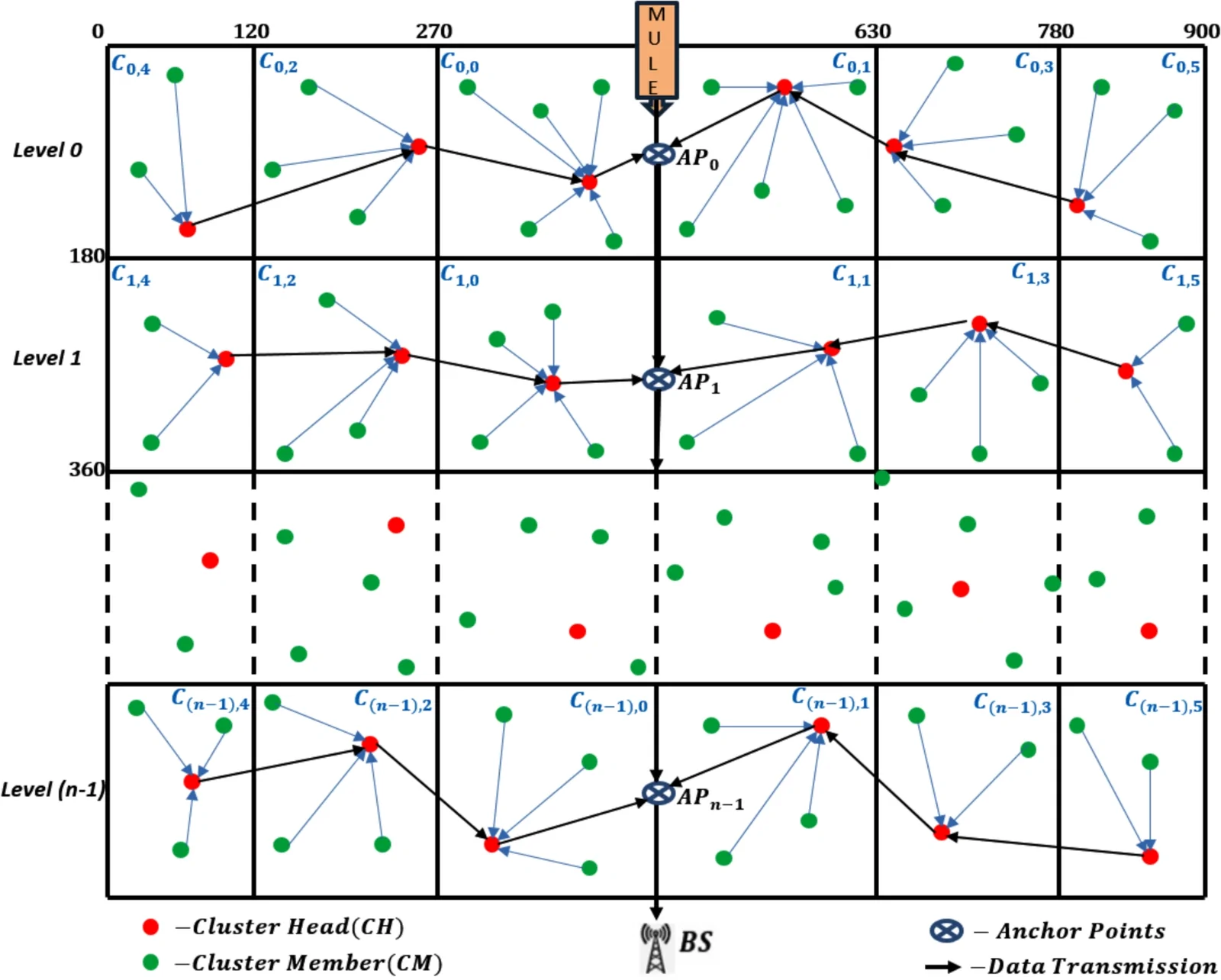
Network data transmission and AP positions for MDC stoppage.

During data collection by the MDC, all the CHs transmit their cluster’s aggregated data received from the member nodes to their predefined neighbor, forwarding the CH towards APs. The data transmission from CH to the forwarding CH and from a CH of one level to an MDC is performed based on the three handshake protocols discussed in the data forwarding section. The total time required to transmit the data for a borderline CH to the nearest CH to AP in the same level is $t_{CH}*numCL/2$ Sec, and in the upper and lower levels, CH requires time in $t_{CH}*(numCL/2 + 1)$ Sec. When all data of a level reaches the last CH nearest to the AP of that level, the CH transfers its aggregated data to the MDC. The MDC maintains a constant velocity while traversing the network’s middle path. Its trajectory remains fixed, following a straight path as depicted in Fig. 5. Along this path, predetermined access points (APs) are positioned at each level where the MDC pauses to gather data from the CHs of each level. Upon reaching the range of the BS, the MDC transfers its accumulated data to the BS. Figure 4 depicts the complete operation of the proposed protocol through a detailed flowchart diagram.

- Larger grid size, *→* fewer cluster heads (CHs) *→* higher transmission cost, and *→* reduced network lifetime.
- Smaller grid size *→* increased the number of CHs *→* higher control overhead but improved load balancing.
- Optimal grid width is given by:
  24 $$\begin{aligned} w = \frac{D_0}{\sqrt{5}} \end{aligned}$$

- A deterministic vertical path eliminates redundant movement of the mobile data collector (MDC).
- It guarantees predictable visitation of cluster heads (CHs).
- It minimizes buffering delay at the CHs.
- It ensures fresh and timely data delivery to the access point (AP).

Effect of MDC velocity:

- Increasing MDC velocity significantly reduces buffer delay at the access point (AP).
- MDC velocity has minimal impact on energy consumption, as each sensor node transmits only once per data collection round.

## Simulation results

This section discusses the simulation parameters used for our scheme and three other schemes. It will also cover the position of APs and the CH change factor (CHC) for CH rotation. The simulation results are analyzed using various performance parameters, including the number of rounds, the number of dead nodes, and the residual energy. These performance metrics will be discussed in two different scenarios of deployed nodes, along with comparison graphs of three existing protocols.

### Simulation setup

**Fig. 6:**
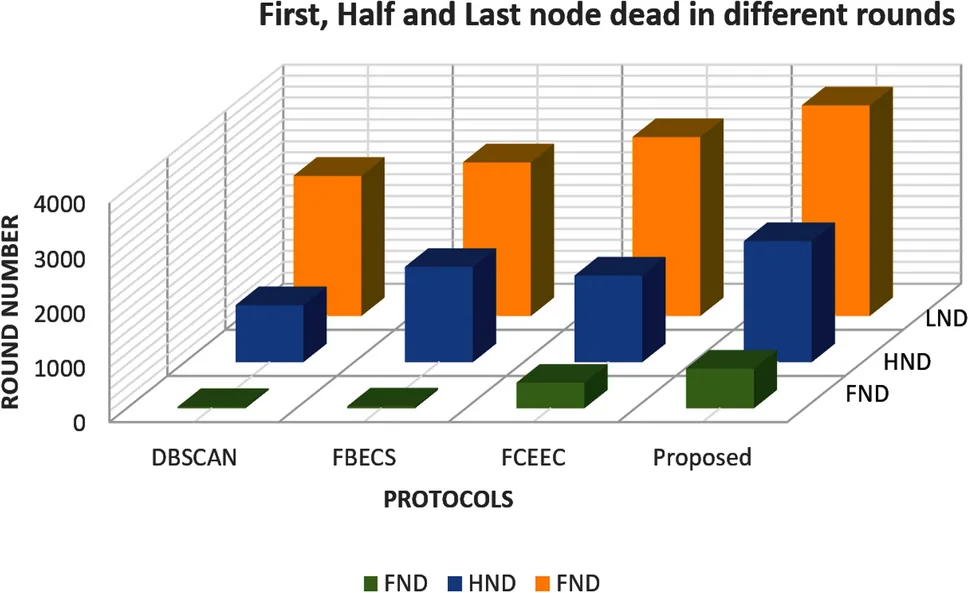
FND, HND, and LND in 1200 nodes.

We conducted simulations of our scheme using OMNet++^35^ on the Windows 10 platform. The deployment area is a 500 *×* 500 square meter region. We have randomly deployed 1200 and 600 nodes to obtain the results. Each node has an initial energy of 1 Joule. The data and control packets have lengths of 1000 bits and 100 bits, respectively. The constant values for E$_{e}$, e$_{fsf}$, and e$_{mpf}$ are 50 nJ/bit, 10 pJ/bit/m$^2$, and 0.0013 pJ/bit/m$^4$, respectively. In our simulation, we placed the APs in the middle section of the network. Table 3 illustrates the simulation parameters used for the simulation. Figures 5 and 6 are created in Microsoft Excel using data obtained from the OMNeT++ simulation tool. We also simulated three similar protocols: (i) FBECS^36^, (ii) DBSCAN^37^, and (iii) FCEEC^38^ with the same parameters for comparison. The simulation results are discussed in the next section.

**Table 3:** Simulation parameters.

Parameter	Value
Simulation platform	OMNet++
Network area	*500 × 500* m$^2$
Threshold distance	87 m
Number of deployed nodes	1200 and 600
Initial energy of node	1 J
$E_{e}$	50 nJ/bit
$e_{fsf}$	10 pJ/bit/m$^2$
$e_{mpf}$	0.0013 pJ/bit/m$^4$
Data packet length	1000 bits
Control packet length	100 bits

### Positions of APs

The APs serve as a designated location where the MDC stops to collect data from the CHs. In our work, we have defined a specific trajectory path for the MDC. This predefined path describes the movement of MDC in a vertical path at the middle section of the network. The positions of the APs in this scenario are indicated by blue solid circles as shown in Figure 5.

### Comparison table for dead nodes in different rounds

**Fig. 7:**
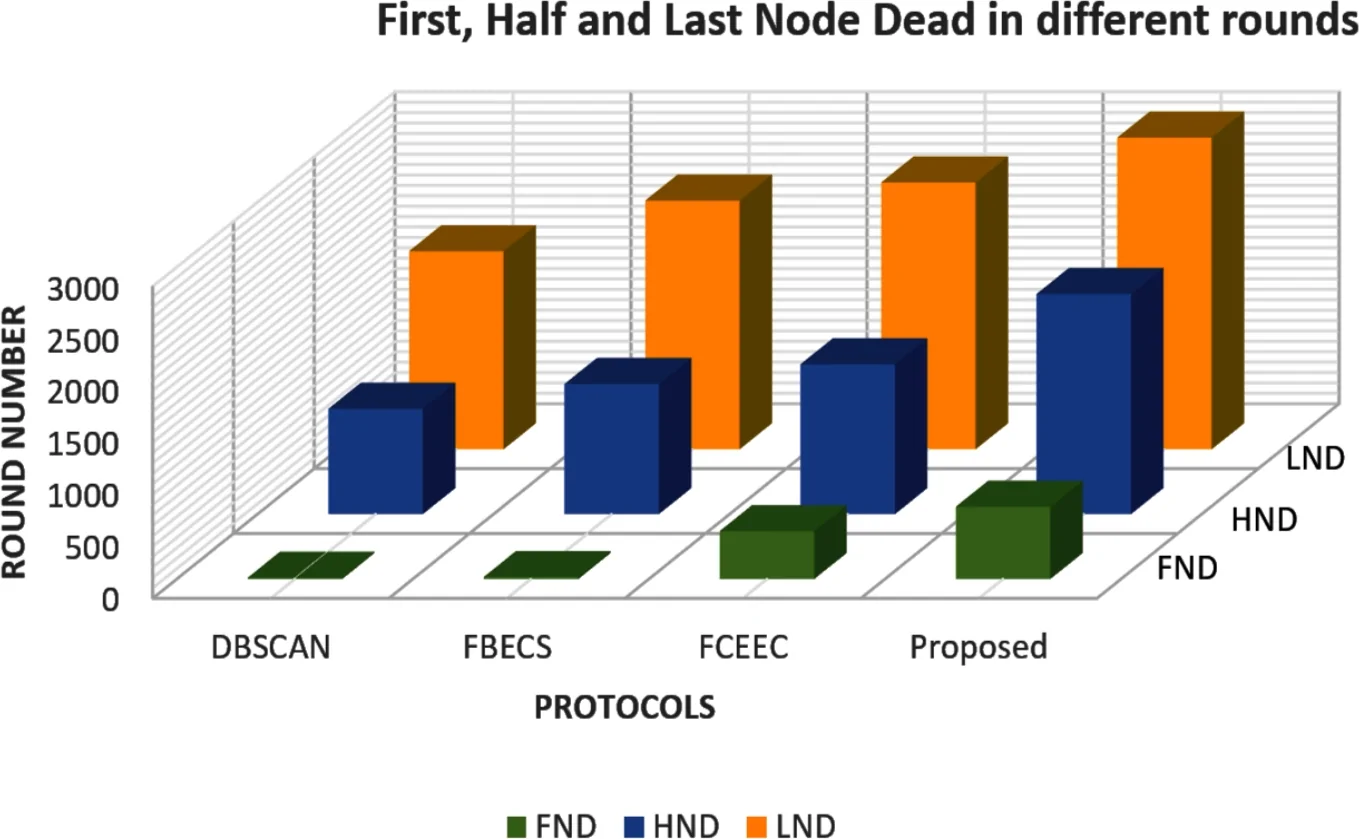
FND, HND, and LND in 600 nodes.

We have evaluated the performance with 1200 and 600 nodes using key parameters: first node dies (FND), half node dies (HND), and last node dies (LND). Along with our proposed scheme, we simulated three other schemes for comparison. The results for these parameters are summarized in Tables 4 and 5 for both node deployment scenarios. Upon analyzing the data presented in Tables 4, 5, Figs. 1, 6 and 7, it becomes evident that our scheme surpasses the other three schemes. Our scheme demonstrates superior performance by sustaining operations for the maximum number of rounds, and the first node dies at 765 rounds, which is notably better than the alternatives.

**Table 4:** FND, HND and LND in different rounds for 1200 nodes.

Schemes	FND	HND	LND
DBSCAN	24	1043	2558
FBECS	32	1438	2806
FCEEC	467	1582	3258
Proposed	724	2217	3832

**Table 5:** FND, HND, and LND in different rounds for 600 nodes.

Schemes	FND	HND	LND
DBSCAN	16	1019	1913
FBECS	15	1264	2386
FCEEC	462	1451	2586
Proposed	694	2104	2983

**Fig. 8:**
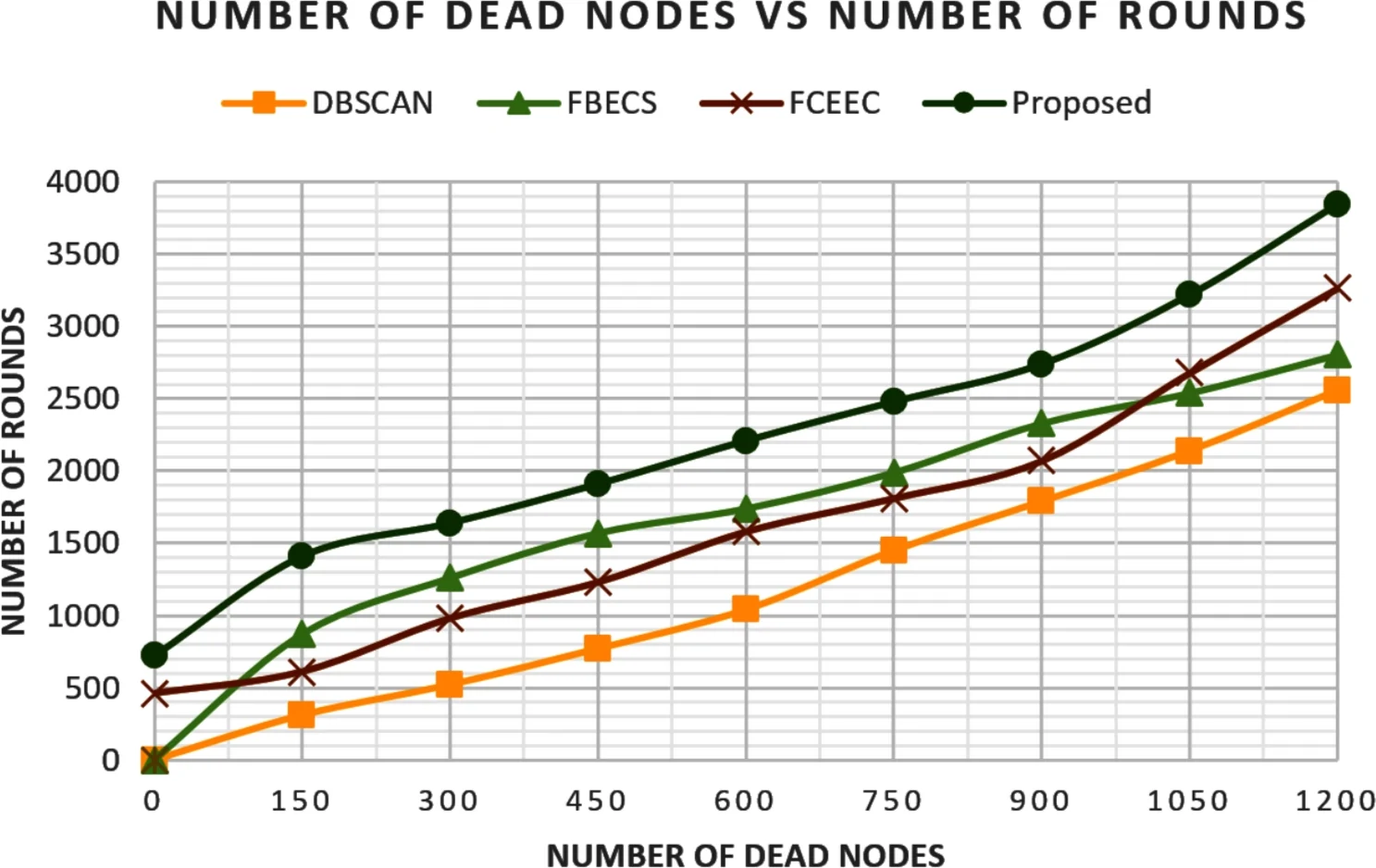
Dead nodes vs round numbers in 1200 nodes.

**Fig. 9:**
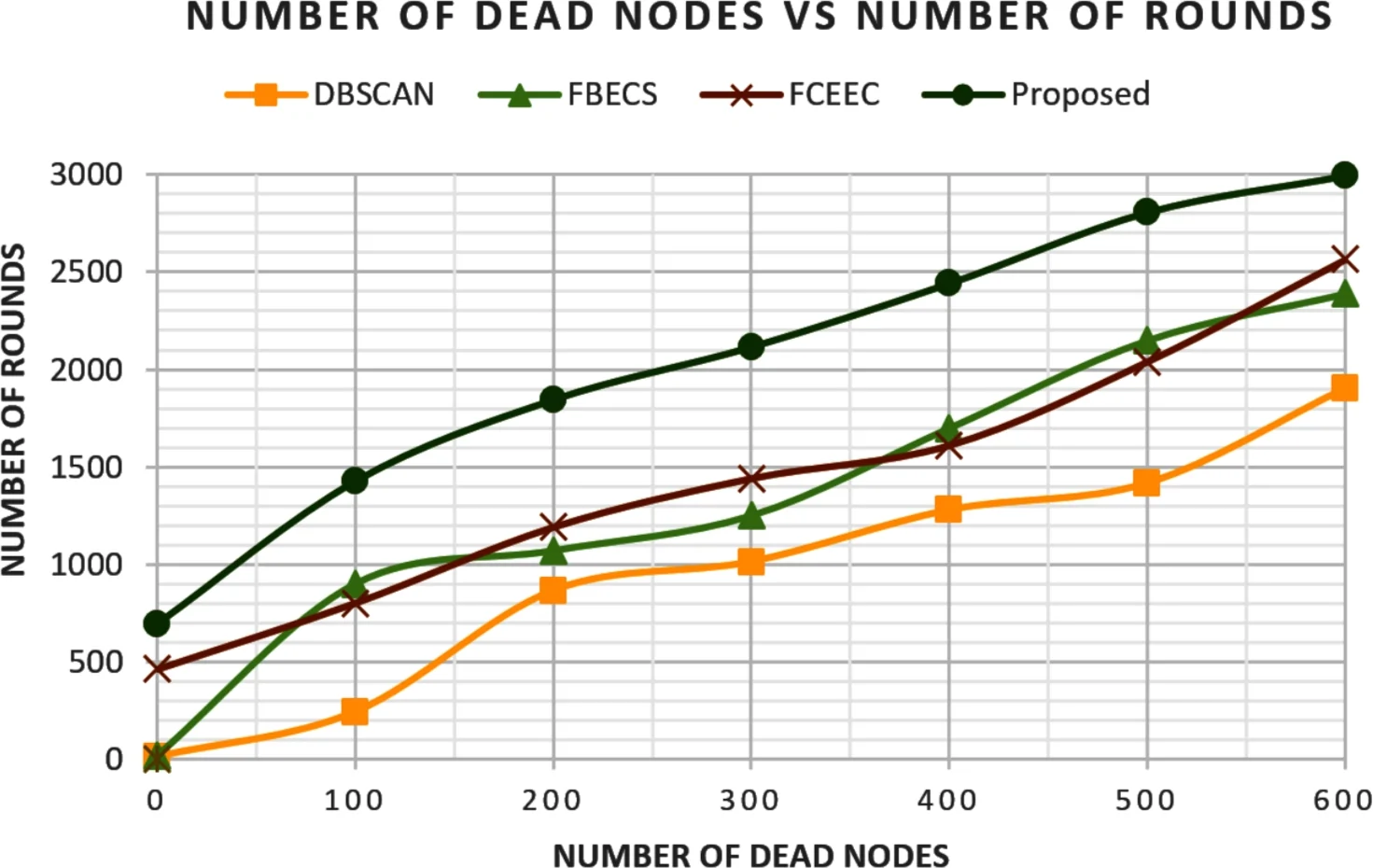
Dead nodes vs round numbers in 600 nodes.

**Fig. 10:**
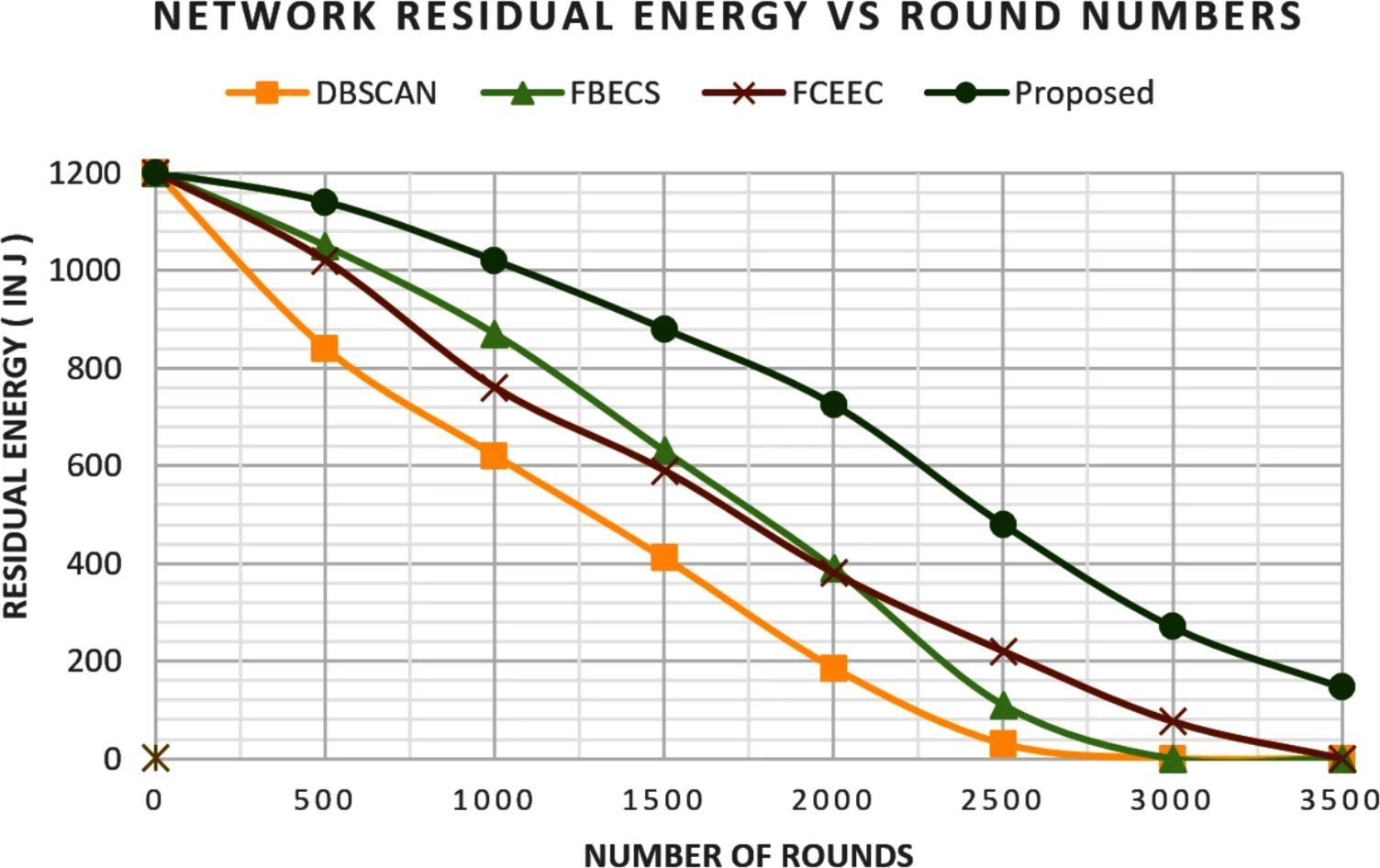
Residual energy vs round numbers in 1200 nodes.

**Fig. 11:**
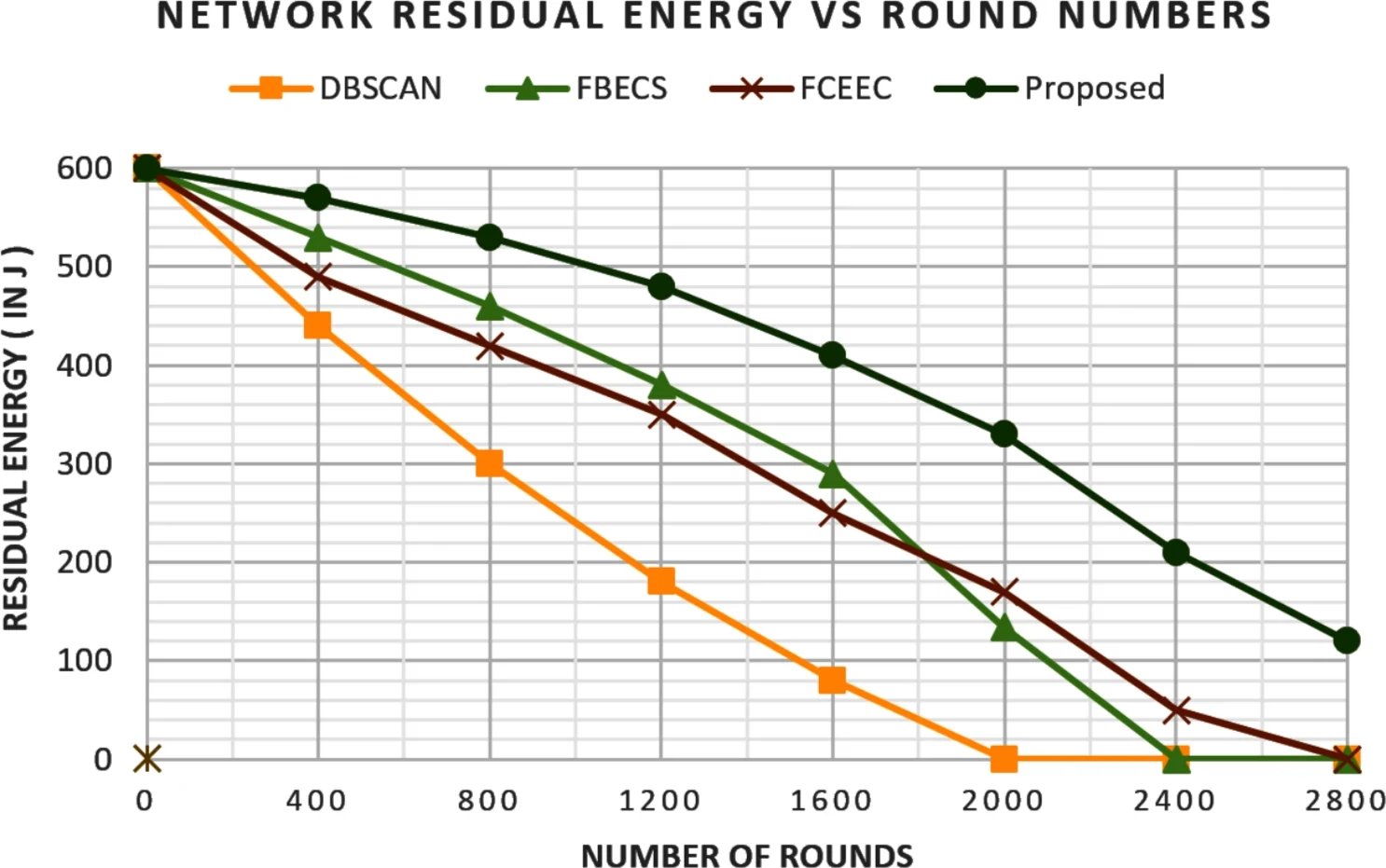
Residual energy vs round numbers in 600 nodes.

### Performance evaluation based on dead nodes and residual energy

Figures 8 and 9 illustrate graphs that display the relationship between the number of rounds and the number of dead nodes in deployments consisting of 1200 and 600 sensor nodes, respectively. We have compared the outcomes of our proposed protocol with those of existing protocols such as FBECS, DBSCAN and FCEEC. As evident from Figs. 8 and 9, our proposed protocol exhibits significantly fewer dead nodes across various rounds compared to the other schemes. Our scheme outperforms in terms of dead nodes in both scenarios: dense deployment of 1200 nodes and light deployment of 600 nodes. This superior performance can be attributed to the CH change factor and the size of the CH. The unequal clustering plays a crucial role in balancing the energy consumption of the nodes by the uniform distribution of the overhead CH role. Consequently, nodes closer to the base station (BS) experience a longer lifespan. This mitigates the energy hole problem, which restricts transmission distance to a threshold value, thereby conserving the transmission energy of the nodes and prolonging the network’s lifetime. Figures 10 and 11 depict the residual energy of the network after each round, considering the deployment of 1200 and 600 nodes, respectively. The comparison in these figures demonstrates that the residual energy of the network surpasses that of the existing protocols in both deployment scenarios in any round. This can be attributed to the fact that fewer nodes become inactive in the corresponding round. The efficient selection of cluster size and the data collection process facilitated by the MDC play a crucial role in achieving this outcome. By enabling direct data collection from CHs, the MDC effectively reduces the number of long-distance transmissions, resulting in substantial energy savings.

## Conclusions and future works

There are various reasons for the better performance of the proposed protocol over existing protocols. It uses an unequal clustering approach, where the size of the clusters near the BS is larger than that of the far-away clusters. The larger cluster is associated with a greater number of nodes, resulting from the higher number of nodes participating in the CH election. This has improved the lifetime of these nodes, resulting in a reduction in the occurrence of energy holes. Due to the centralized nature of the proposed protocol, sensor nodes play a minimal role in selecting the CH and discovering routes. This minimizes energy consumption for nodes tasked with overhead duties. The protocol restricts the transmission range for any node in the network to $D_o$ to ensure the energy depletion of the sensor node at a rate of $d^2$, which is remarkably less than $d^4$. The protocol ensures uniform energy consumption and data traffic in the entire network, resulting in a reduction of energy hole problems. The use of MDC in the protocol conserves the energy of nodes near APs, thereby extending the life of the nearer nodes. The change of CH based on the CH change factor (CHC) balances the energy consumption of nodes throughout the network. All these factors conserve the energy of sensor nodes, which provides a longer network lifespan and a more stable network. The simulation results demonstrate that the lifetime of the entire network is approximately 3200 rounds, showcasing the superiority of our scheme over other approaches.

In the future, we will try to analyze the other movement patterns of the MDC with different speeds in order to increase network lifespan to a much greater extent.

## Data Availability

All data generated or analyzed during this study are included in this published article.

## References

[1] Akyildiz, I. F., Su, W., Sankarasubramaniam, Y. & Cayirci, E. Wireless sensor networks: A survey. *Comput. Netw.* **38**(4), 393–422 (2002).

[2] Rashid, B. & Rehmani, M. H. Applications of wireless sensor networks for urban areas: A survey. *J. Netw. Comput. Appl.* **60**, 192–219 (2016) http://www.sciencedirect.com/science/article/pii/S1084804515002702.

[3] Perillo, M., Cheng, Z. & Heinzelman, W. An analysis of strategies for mitigating the sensor network hot spot problem. In *The Second Annual International Conference on Mobile and Ubiquitous Systems: Networking and Services*, 474–478 (2005).

[4] Pantazis, N. A., Nikolidakis, S. A. & Vergados, D. D. Energy-efficient routing protocols in wireless sensor networks: A survey. *IEEE Commun. Surv. Tutor.* **15**(2), 551–591 (2013).

[5] Heinzelman, W. R., Chandrakasan, A. & Balakrishnan, H. Energy-efficient communication protocol for wireless microsensor networks. In *System Sciences, 2000. Proceedings of the 33rd annual Hawaii international conference on*, 01–10 (IEEE, 2000).

[6] Afsar, M. M. & Tayarani-N, M.-H. Clustering in sensor networks: A literature survey. *J. Netw. Comput. Appl.* **46**, 198–226 (2014).

[7] Anastasi, G., Conti, M., Di Francesco, M. & Passarella, A. Energy conservation in wireless sensor networks: A survey. *Ad Hoc Netw.* **7**(3), 537–568 (2009).

[8] Singh, S. K., Kumar, P. & Singh, J. P. A survey on successors of leach protocol. *IEEE Access* **5**, 4298–4328 (2017).

[9] Arjunan, S. & Pothula, S. A survey on unequal clustering protocols in wireless sensor networks. *J. King Saud Univ. Comput. Inf. Sci.* http://www.sciencedirect.com/science/article/pii/S1319157817300927 (2017).

[10] Gupta, V. & Pandey, R. An improved energy aware distributed unequal clustering protocol for heterogeneous wireless sensor networks. *Eng. Sci. Technol. Int. J.* **19**(2), 1050–1058 (2016) http://www.sciencedirect.com/science/article/pii/S2215098616000045.

[11] Singh, S., Malik, A. & Kumar, R. Energy efficient heterogeneous DEEC protocol for enhancing lifetime in WSNS. *Eng. Sci. Technol. Int. J.* **20**(1), 345–353 (2017) http://www.sciencedirect.com/science/article/pii/S2215098616301562.

[12] Singh, S. Energy efficient multilevel network model for heterogeneous WSNS. *Eng. Sci. Technol. Int. J.* **20**(1), 105–115 (2017) http://www.sciencedirect.com/science/article/pii/S2215098616303470.

[13] Zadeh, P. H., Schlegel, C. & MacGregor, M. H. Distributed optimal dynamic base station positioning in wireless sensor networks. *Comput. Netw.* **56**(1), 34–49 (2012).

[14] Osama Moh’d Alia. Dynamic relocation of mobile base station in wireless sensor networks using a cluster-based harmony search algorithm. *Inf. Sci.* **385**, 76–95 (2017).

[15] Joon, R., Tomar, P., Kumar, G., Balusamy, B. & Nayyar, A. Unequal clustering energy hole avoidance (UCEHA) algorithm in cognitive radio wireless sensor networks (CRWSNS). *Wirel. Netw.* **31**(1), 735–757 (2025).

[16] Sharmin, N., Karmaker, A., Lambert, W. L., Alam, M. S. & Shawkat, M. S. A. Minimizing the energy hole problem in wireless sensor networks: A wedge merging approach. *Sensors* **20**(1), 277 (2020). 31947840 10.3390/s20010277 PMC6983154

[17] Heinzelman, W. B., Chandrakasan, A. P. & Balakrishnan, H. An application-specific protocol architecture for wireless microsensor networks. *IEEE Trans. Wirel. Commun.* **1**(4), 660–670 (2002).

[18] Qiang, T., Bingwen, W. & Zhicheng, D. Ms-leach: A routing protocol combining multi-hop transmissions and single-hop transmissions. In *Pacific-Asia Conference on Circuits, Communications and Systems*, 107–110 (IEEE, 2009).

[19] Biradar, R. V., Sawant, S. R., Mudholkar, R. R. & Patil, V. C. Multihop routing in self-organizing wireless sensor networks. *Int. J. Comput. Sci. Issues (IJCSI)* **8**(1), 155–164 (2011).

[20] Antoo, A. & Mohammed, A. R. Eem-leach: energy efficient multi-hop leach routing protocol for clustered wsns. In *2014 Intl Conf on Control, Instrumentation, Communication and Computational Technologies (ICCICCT)*, 812–818 (IEEE, 2014).

[21] Liu, W.-d., Wang, Z.-d., Zhang, S. & Wang, Q.-q. A low power grid-based cluster routing algorithm of wireless sensor networks. In *2010 Intl Forum on Information Technology and Applications (IFITA)*, vol. 1, 227–229 (IEEE, 2010).

[22] Amrutha, K.M., Ashwini, P., Raj, D. K., Rani, G. K. & Mundada, M. R. Energy efficient clustering and grid based routing in wireless sensor networks. In *Intl Conf on Advances in Computing*, 69–74 (Springer, 2013).

[23] Jannu, S. & Jana, P. K. A grid based clustering and routing algorithm for solving hot spot problem in wireless sensor networks. *Wirel. Netw.* 1–16 (2015).

[24] Shah, R.C., Roy, S., Jain, S. & Brunette, W. Data mules: modeling a three-tier architecture for sparse sensor networks. In *Sensor Network Protocols and Applications, 2003. Proceedings of the First IEEE. 2003 IEEE International Workshop on*, 30–41 (2003).

[25] Singh, S. K., Kumar, P. & Singh, J. P. An energy efficient odd-even round number based data collection using mules in wsns. In *2016 International Conference on Wireless Communications, Signal Processing and Networking (WiSPNET)*, 1255–1259 (2016).

[26] Nitesh, K., Kaswan, A. & Jana, P. K. Energy density based mobile sink trajectory in wireless sensor networks. *Microsyst. Technol.*10.1007/s00542-017-3569-4 (2017).

[27] Singh, S. K., Kumar, P. & Singh, J. P. An energy efficient protocol to mitigate hot spot problem using unequal clustering in WSN. *Wirel. Pers. Commun.*10.1007/s11277-018-5716-3 (2018).

[28] Chen, S., Huang, Q., Zhang, Y. & Li, X. Double sink energy hole avoidance strategy for wireless sensor network. *EURASIP J. Wirel. Commun. Netw.* **2020**(1), 226 (2020).

[29] Dogra, R. et al. A novel dynamic clustering approach for energy hole mitigation in internet of things-based wireless sensor network. *Int. J. Commun. Syst.* **34**(9), e4806 (2021).

[30] Li, J., Han, Q. & Wang, W. Characteristics analysis and suppression strategy of energy hole in wireless sensor networks. *Ad Hoc Netw.* **135**, 102938 (2022).

[31] Edla, D. R., Lipare, A. & Parne, S. R. Load balanced cluster formation to avoid energy hole problem in WSN using fuzzy rule-based system. *Wirel. Netw.* **29**(3), 1299–1310 (2023).

[32] Vahabi, S., Mojab, S. P., Eslaminejad, M. & Dashti, S. E. Eam: energy aware method for chain-based routing in wireless sensor network. *J. Ambient. Intell. Humaniz. Comput.* **13**(9), 4265–4277 (2022).

[33] Vahabi, S., Mojab, S. P., Hozhabri, A. & Daneshvar, A. Reinforcement learning movement path for multiple mobile sinks in wireless sensor networks. *Int. J. Commun Syst* **36**(6), e5402 (2023).

[34] Lindsey, S. & Raghavendra, C. S. Pegasis: Power-efficient gathering in sensor information systems. In *Proceedings, IEEE Aerospace Conference*, vol. 3, 3 (IEEE, 2002).

[35] Varga, A. Omnet++. In *Modeling and Tools for Network Simulation*, 35–59 (Springer, 2010).

[36] Mehra, P. S., Doja, M. N. & Alam, B. Fuzzy-based enhanced cluster head selection (FBECS) for WSN. *J. King Saud Univ.-Sci.* **32**(1), 390–401 (2020).

[37] Sahoo, L., Sen, S., Tiwary, K. S., Moslem, S. & Senapati, T. Improvement of wireless sensor network lifetime via intelligent clustering under uncertainty. *IEEE Access* (2024).

[38] Jagan, G. & Jayarin, P. J. Wireless sensor network cluster head selection and short routing using energy efficient electrostatic discharge algorithm. *J. Eng.* **2022**, 1–10 (2022).

